# Involvement of *E. coli* 6S RNA in Oxidative Stress Response

**DOI:** 10.3390/ijms23073653

**Published:** 2022-03-26

**Authors:** Olga Y. Burenina, Daria A. Elkina, Anna Ovcharenko, Valeria A. Bannikova, M. Amri C. Schlüter, Tatiana S. Oretskaya, Roland K. Hartmann, Elena A. Kubareva

**Affiliations:** 1 Center of Life Sciences, Skolkovo Institute of Science and Technology, 121205 Moscow, Russia; 2 Department of Chemistry, Lomonosov Moscow State University, 119991 Moscow, Russia; anna.ovcharenko@wwu.de (A.O.); bannikova.valeria@yandex.ru (V.A.B.); 3 Belozersky Institute of Physico-Chemical Biology, Moscow State University, 119991 Moscow, Russia; yolkinada@gmail.com (D.A.E.); oretskaya@belozersky.msu.ru (T.S.O.); kubareva@belozersky.msu.ru (E.A.K.); 4 Institute of Pharmaceutical Chemistry, Philipps-University Marburg, 35037 Marburg, Germany; amri.schlueter@pharmazie.uni-marburg.de (M.A.C.S.); roland.hartmann@staff.uni-marburg.de (R.K.H.)

**Keywords:** small non-coding RNAs, 6S RNA, RNA polymerase, regulation of transcription, bacterial oxidative stress response

## Abstract

6S RNA, a small non-coding RNA present in almost all bacteria, inhibits transcription via direct binding to RNA polymerase holoenzymes. The mechanism of 6S RNA action was investigated to a large extent in *E. coli*, however, lack of 6S RNA (Δ*ssrS*) was demonstrated to be unfavorable but not essential for cell survival under various growth conditions. In the present study, we revealed, for the first time, a lethal phenotype of the Δ*ssrS* strain in the presence of high concentrations of H_2_O_2_. This phenotype was rescued by complementation of the *ssrS* gene on a plasmid. We performed comparative qRT-PCR analyses on an enlarged set of mRNAs of genes associated with the oxidative stress response, allowing us to identify four genes known to be involved in this pathway (*soxS*, *ahpC*, *sodA* and *tpx*) that had decreased mRNA levels in the Δ*ssrS* strain. Finally, we performed comparative proteomic analyses of the wild-type and Δ*ssrS* strains, confirming that Δ*ssrS* bacteria have reduced levels of the proteins AhpC and Tpx involved in H_2_O_2_ reduction. Our findings substantiate the crucial role of the riboregulator 6S RNA for bacterial coping with extreme stresses.

## 1. Introduction

Regulation of transcription is a central mechanism in prokaryotes for adjusting gene expression to changes, in particular, under unfavorable environmental conditions [1,2,3]. In natural habitats, bacteria strive for survival under nutrient starvation, need to adapt quickly to, e.g., fluctuations in temperature, pressure, humidity, pH and osmotic strength, adjust their metabolism according to the density of the population, or differentiate into subpopulations for the sake of species survival [4,5,6]. Fast switching to an expression of stress response proteins is widely achieved by activating the transcription of specific sets of mRNAs, processes that are not only regulated by proteinogenic transcription factors [7] but also by small non-coding RNAs (ncRNAs) [8,9,10]. Small ncRNAs have lengths ≤ 200 nt and usually do not participate in translation. To date, just a few examples of bifunctional RNAs are known in bacteria that act both as riboregulator and mRNA, such as RNAIII and Psm-mec RNA from *Staphylococcus aureus*, SgrS RNA from *Escherichia coli* and SR1 RNA from *Bacillus subtilis* [11]. To date, dozens of ncRNAs are known to play various regulatory roles in bacteria via binding to complementary mRNAs or by direct interaction with proteins [12,13]. The high impact of some ncRNAs on the regulation of gene expression and successful bacterial growth also makes them prospective targets for industrial applications [14].

6S RNA is an abundant bacterial ncRNA that directly binds to RNA polymerase (RNAP) holoenzymes, particularly in the stationary phase of cell growth [15,16]. The resulting inhibition of transcription entails global changes in the bacterial transcriptome [17,18] and proteome [19]. Although 6S RNA genes are found in the majority of bacterial species [20], detailed functional studies have focused mainly on 6S RNAs from *E. coli* and *B. subtilis*. Historically, the first documented phenotype of an *E. coli* 6S RNA knockout (Δ*ssrS*) strain was reduced survival compared to the parental strain under conditions of extended cultivation (20 days) and in competitive cultivation experiments [21]. *E. coli* 6S RNA was shown to regulate the expression of various transcription and translation factors, transporters and enzymes involved in the metabolism of purines and degradation of amino acids. Notably, a number of stress-related genes were also shown to be dysregulated in Δ*ssrS* cells, especially in the stationary phase, in particular, *asr* (acid shock protein), *dps* (DNA protection protein), *cspA* and *yfiA* (cold shock proteins), *sra* and *smpB* (ribosome stabilizing proteins), as well as *uspG* and *uspF* (general stress proteins) [17]. The *E. coli* 6S RNA knockout also caused derepression of the *relA* gene encoding ppGpp synthase I in the early stationary phase, resulting in decreased rRNA expression and enhanced expression of genes involved in amino acid biosynthesis [22]. A pronounced effect of 6S RNA depletion was observed in the stationary phase under stringent alkaline conditions (pH 9.3). The presence of 6S RNA normally ensures controlled expression of the transcription factor PspF that is required for expression of the *pspABCDE* and *pspG* operons involved in alkaline stress response. Upon 6S RNA knockout, these proteins are overexpressed and improve stationary cell survival under high pH conditions [23].

Similar and overlapping phenotypes were observed for the Gram-positive *B. subtilis*, although this bacterium is quite different in terms of natural habitats, physiology and metabolism. *B. subtilis* and other Firmicutes express two different 6S RNAs (6S-1 and 6S-2) [24]. Deletion of both 6S RNAs in the laboratory strain PY79 also caused a decrease of *B. subtilis* culture density in the late stationary phase (24 h cultivation) and was advantageous under extremely alkaline conditions (pH 9.8) [19]. Additionally, the lack of *B. subtilis* 6S-1 RNA (the assumed functional homolog of *E. coli* 6S RNA) in the strain 168 caused a delay in the outgrowth of cell cultures from the stationary phase and resulted in earlier sporulation [25,26]. Proteome analyses of *B. subtilis* PY79 derivative strains with knockouts of genes encoding its two 6S RNA paralogs, 6S-1 and 6S-2 RNA, also revealed changes in expression levels of many proteins involved in metabolisms of purines, amino acids and carbohydrates, including a number of stress response regulators, such as *guaB*, *cysK*, *tpx* (superoxide-inducible proteins), *yvyD* and *yjlD* (glucose starvation-inducible proteins), *rplJ* (cold shock and salt stress protein), *greA*, *yraA*, *ahpC*, *sodA* and *nadE* (general stress proteins) [19]. Recently *B. subtilis* 6S-2 RNA, but not 6S-1 RNA, was demonstrated to regulate biofilm formation, swarming activity and sporulation in the undomesticated wild-type strain NCIB 3610 [27], phenotypes that were not observable in 6S RNA knockout strains derived from the laboratory strain PY79 [19]. Noteworthy, and in contrast to the 6S RNA double knockout in the *B. subtilis* PY79 background (see above), deletion of both 6S RNA genes in strain NCIB 3610 retarded outgrowth under alkaline stress (pH 9.5) relative to the parental and single knockout derivative strains [27].

Evidence for a key role of 6S RNA in the regulation of bacterial stress responses has also been growing owing to studies in other bacteria beyond *E. coli* and *B. subtilis*. For example, lack of 6S RNA was reported to lead to slower growth of *Rhodobacter sphaeroides* under high salt stress (250 mM NaCl) [28], to delay recovery of *Synechocystis* sp. PCC 6803 cells from nitrogen starvation [29] and to reduce survival of *Salmonella enterica* serovar Typhimurium under acidic stress (pH 3.0) [30]. Moreover, in the latter case, the knockout strain had a reduced ability to invade HeLa cells and showed attenuated virulence in a mouse model [30]. Similar reductions of bacterial pathogenicity upon 6S RNA gene deletion were observed for *L. pneumophila* during infection of the protist *Acanthamoeba castellanii* or mammalian THP-1 cells [31], and for the Lyme disease-causing spirochete *Borrelia burgdorferi* in a mouse infection model [32].

In summary, 6S RNA functions seem to be quite diverse across different bacterial phyla, possibly species-specific in some cases. Yet, an emerging commonality of 6S RNAs is their importance for bacterial physiology, especially under adverse environmental conditions. Surprisingly, 6S RNA knockouts have so far not been reported to be lethal for their bacterial host, despite the RNA’s function as a global regulator of transcription. Seemingly, 6S RNAs are intertwined with additional, partly redundant mechanisms of gene expression control that can compensate for the loss of 6S RNA to some extent. 

In the present work, we observed the involvement of 6S RNA in the oxidative stress response in *E. coli* and report the first-ever lethal phenotype of a 6S RNA-deficient strain at high concentrations of hydrogen peroxide_._ Notably, *E. coli* 6S RNA knockouts have been studied since 1985 [33], but no data for such conditions were reported. Thus, our work has discovered a novel pathway of 6S RNA-mediated regulation of the *E. coli* stress response that is critical for cell survival.

## 2. Results

### 2.1. The Oxidative Stress Phenotype of the 6S RNA-Deficient Strain

**Figure 1:**
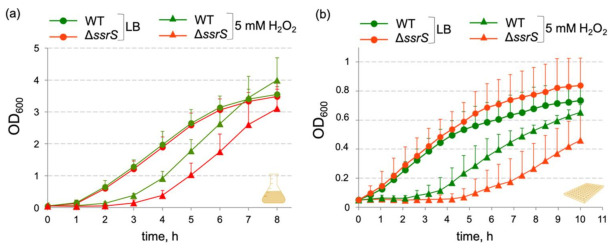
Lack of 6S RNA leads to an extended delay in outgrowth of *E. coli* cell cultures in the presence of 5 mM H_2_O_2_. Growth curves of *E. coli* MG1655 WT (green symbols) and Δ*ssrS* bacteria (red symbols) in LB medium in the absence or presence of 5 mM H_2_O_2_. Cells were either grown in liquid culture flasks (**a**) or in a 96-well microtiter plate format (**b**) with manual monitoring of optical density at 600 nm (OD_600_) in three and six biological replicates, respectively.

We previously constructed an *E. coli* MG1655 Δ*ssrS* strain where we replaced the 6S RNA gene (*ssrS*) with a kanamycin resistance cassette [34]. In the course of phenotypic strain analyses, we noticed an effect of *E. coli* 6S RNA gene deletion on cell growth and survival under oxidative stress conditions. Upon inoculation of fresh medium containing 5 mM H_2_O_2_ with stationary phase cells (overnight culture), we observed an extended delay in outgrowth for the Δ*ssrS* strain relative to the parental MG1655 (wild type, WT) strain (Figure 1).

This relative outgrowth delay reached 1 to 2 h (Figure 1) or even up to 4 h in other experiments (Figure S1). The exact period of the lag phase varied between individual experiments, possibly due to fluctuations in media preparations that might affect the rate of H_2_O_2_ reduction. The phenotype was reproducibly observed for growth in liquid culture flasks (Figure 1a) and 96-well plates (Figure 1b) despite the differences between these two setups, including medium volume, stirring speed and aeration. In the presence of 2 mM H_2_O_2_ we observed the same effect, although the lag phase extension of the Δ*ssrS* strain tended to be somewhat shorter (~ 1 h; Figure S2). The presence of 10 mM H_2_O_2_ caused very long (>12 h) outgrowth delays for both, the WT and Δ*ssrS* strain, with increasing variations between replicates (data not shown), thus excluding the calculation of meaningful average growth curves.

**Figure 2:**
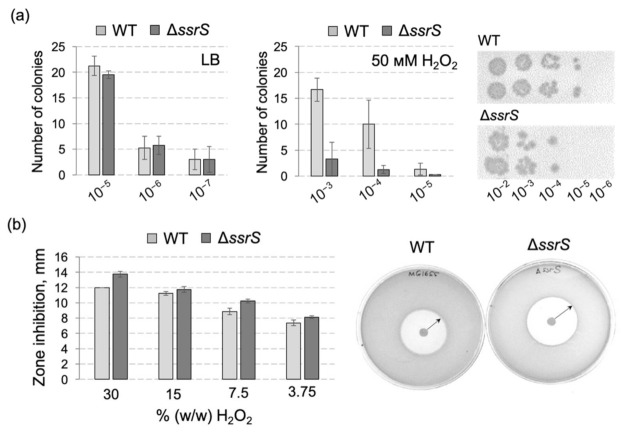
The lack of 6S RNA leads to reduced survival of *E. coli* MG1655 cells in the presence of H_2_O_2_. (**a**) Results of cell culture plating (serial dilutions indicated on the X-axis) after 30 min of incubation in the absence (left panel; LB control) or presence of 50 mM H_2_O_2_ (central panel), 4 biological replicates in each case. A plating example after culture incubation with 50 mM H_2_O_2_ is shown on the right. (**b**) Results of the zone of inhibition test for *E. coli* MG1655 WT and Δ*ssrS* bacteria grown in the presence of different H_2_O_2_ concentrations (left panel, based on three biological replicates each); corresponding example agar plates are illustrated on the right: after streaking of cell culture dilution, a paper disk (5 mm diameter) soaked with 30% (*w/w*) H_2_O_2_ was placed in the center (gray sphere) of the plate; the white area is the zone of no growth whose diameter was measured with a ruler after 24 h at 37 °C; 30% (*w/w*) H_2_O_2_ corresponds to 9.79 M, 3.75% to 1.22 M.

To explore whether retarded growth of the knockout strain resulted from a higher frequency of cell death, we analyzed the number of survivor colonies after H_2_O_2_ treatment by plating of serial culture dilutions. To further accentuate the difference between the two strains, we increased the concentration of H_2_O_2_ in the culture medium to 50 mM and withdrew culture aliquots just after 30 min of incubation. In these experiments, Δ*ssrS* bacteria showed at least 10-fold lower viability than the parental WT strain (Figure 2a). We additionally tested the influence of H_2_O_2_ on *E. coli* growth on solid medium by a conventional inhibition zone assay. This also demonstrated a reproducibly higher sensitivity of the Δ*ssrS* strain to H_2_O_2_ (Figure 2b). 

### 2.2. Investigation of E. coli Strains with Complementation of the ssrS Gene

**Figure 3:**
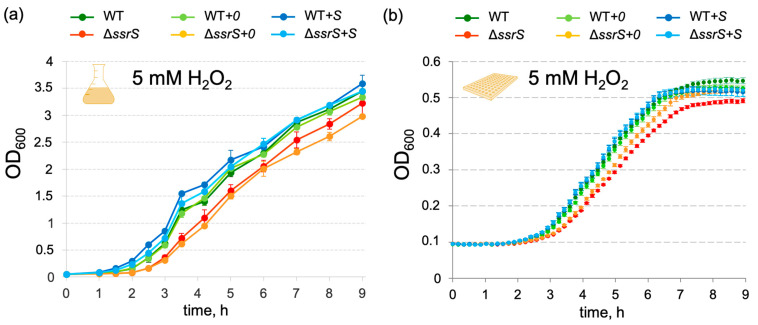
Complementation of the 6S RNA knockout strain by a plasmid-borne *ssrS* gene rescues the growth defect. (**a**) Growth curves of *E. coli* strains in the presence of 5 mM H_2_O_2_ either during cultivation in flasks with manual monitoring of optical density (OD_600_) or (**b**) in 96-well plates using an automated scanning reader; based on 3 biological replicates in each type of experiment.

Considering that *E. coli* 6S RNA has been studied for years without any reported evidence for such an oxidative stress phenotype, we analyzed the phenotype of the MG1655 Δ*ssrS* strain complemented with the wild-type *ssrS* gene expressed from a low-copy plasmid (p177_*ssrS*; see Section 4). The plasmid harbored the *ssrS* gene under the control of its native P1 promoter and the transcription terminator of the *E. coli rnpB* gene (see Supplementary Data, Figures S3–S5, Table S1). The *E. coli* MG1655 WT and Δ*ssrS* strains were either transformed with plasmid p177_*ssrS* or the empty vector (p177_*empty*) resulting in complementation strains abbreviated as WT*+S*, Δ*ssrS+S*, WT*+0* and Δ*ssrS+0*, respectively. Expression of 6S RNA in strains WT, WT*+0*, WT*+S* and Δ*ssrS+S*, as well as the absence of 6S RNA in strains Δ*ssrS* and Δ*ssrS+0*, was confirmed by Northern blot analysis (Figure S6). Stationary phase (24 h) levels of 6S RNA were comparable in strains WT, WT*+0*, WT*+S* and Δ*ssrS+S*, while 6S RNA levels in exponential (2 h) and transition (8 h) phase were somewhat lower in the WT and WT*+0* than in the WT*+S* and Δ*ssrS+S* strains. All strains showed essentially uniform growth behavior in standard rich (LB) medium (Figure S7). Comparative growth analysis in the presence of 5 mM H_2_O_2_ confirmed the retarded outgrowth phenotype for the Δ*ssrS* and Δ*ssrS+0* strains, whereas the Δ*ssrS+S* complementation strain rescued this defect displaying growth behavior indistinguishable from that of the WT, WT*+0* and WT*+S* strains (Figure 3). The same tendency of delay in growth of Δ*ssrS* and Δ*ssrS+0* strains was observed for a number of individual experiments even in the presence of 2 mM H_2_O_2_, although in the latter case the difference between strains was rather small (Figure S8). We also verified the stability of the Δ*ssrS* phenotype in experiments where we started a new outgrowth in the presence of 5 mM H_2_O_2_ from 24 h cultures grown under the same oxidative stress condition (Figure S9). The Δ*ssrS* bacteria showed the outgrowth delay also in this second round of exponential growth, whereas the Δ*ssrS+S* strain behaved as the WT. Thus, the observed phenotype is a functional feature of cells lacking 6S RNA and does not result from spontaneous selection of subpopulations resistant to H_2_O_2_. 

### 2.3. Lack of 6S RNA Is Lethal for E. coli in the Presence of Elevated H_2_O_2_ Concentrations 

**Figure 4:**
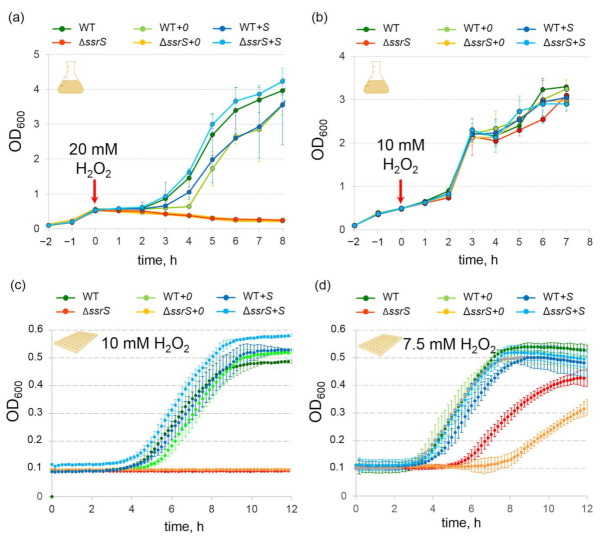
Lack of 6S RNA leads to lethality of *E. coli* in the presence of high concentrations of H_2_O_2_. (**a**,**b**) Growth curves of *E. coli* strains (three biological replicates) in the presence of 20 or 10 mM H_2_O_2_ (f.c.) that were directly added to exponentially growing (OD_600_ ~ 0.5) *E. coli* flask cultures. Optical density was monitored manually. (**c**,**d**) Growth of *E. coli* strains (3 biological replicates) in 96-well plates monitored by an automated scanning reader. Here, *E. coli* strains were grown in LB medium up to an OD_600_ ~ 0.5 in the absence of H_2_O_2_, followed by 1:5 dilution of culture medium containing different amounts of H_2_O_2_ (f.c. 10 or 7.5 mM) before resuming growth and monitoring of optical density.

In the experiments discussed so far, we adjusted fresh media to the respective H_2_O_2_ concentration and then inoculated with an overnight culture grown in standard LB medium. To further characterize the discovered phenotype of 6S RNA-deficient cells, we tested several oxidative stress regimens. The phenotype was exacerbated when 20 mM H_2_O_2_ (f.c.) was added to exponentially growing (OD_600_ ~ 0.5) *E. coli* cell cultures in flasks, resulting in the lethality of Δ*ssrS* and Δ*ssrS+0* bacteria (Figure 4a). When adding only 10 mM H_2_O_2_, the observed effect vanished (Figure 4b). At intermediate H_2_O_2_ concentrations (15 mM and 17.5 mM), the growth delay of the knockout strains correspondingly increased (Figure S10). We were also able to reproduce this effect in the 96-well plate format. Considering the fact that OD_600_ values are not comparable in flask and plate format we slightly modified the protocol. We first grew *E. coli* pre-cultures in flasks in LB medium up to OD_600_ ~ 0.5 (exponential phase) in the absence of H_2_O_2_, and then diluted this culture 1:5 in media containing different amounts of H_2_O_2_; the resulting suspensions were transferred to 96-well plates for growth monitoring. In this type of experiment 20 mM H_2_O_2_ was very toxic to all of the strains, but 10 mM H_2_O_2_ was only lethal for Δ*ssrS* and Δ*ssrS+0* bacteria (Figure 4c and Figure S11). The latter two strains were also considerably delayed in cell growth in the presence of 7.5 mM H_2_O_2_ (Figure 4d). Moreover, survival of Δ*ssrS+0* bacteria was reduced in comparison to Δ*ssrS* bacteria, which may be attributable to the consumption of resources for maintaining the plasmid. Higher H_2_O_2_ sensitivity of cell cultures grown in 96-well plates is likely due to the specific experimental setup, probably resulting in slower H_2_O_2_ reduction owing to less thorough aeration and stirring of media in comparison to culturing in flasks. In some independent experiments, we also observed a clear trend toward faster growth of the Δ*ssrS+S* and WT*+S* strains relative to WT and WT*+0* under H_2_O_2_ stress conditions (Figure S11). This suggests that elevated 6S RNA levels during exponential growth may confer enhanced protection against damage by H_2_O_2_ stress. However, in most cases, this effect was rather small (Figure 4c) and not clearly reproducible in all performed experiments. 

**Figure 5:**
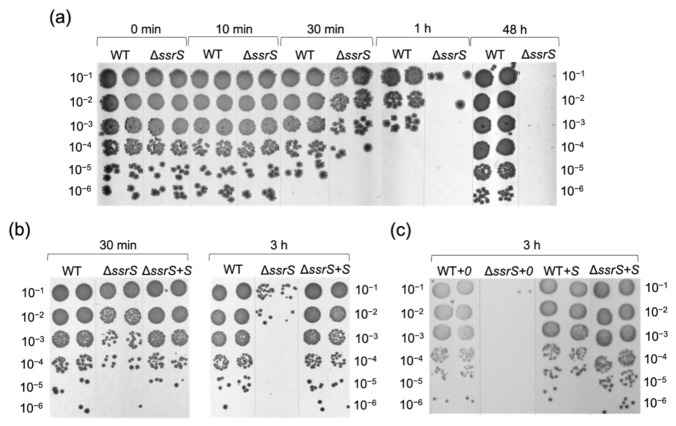
*E. coli* cells with deletion of the *ssrS* gene show decreased viability in the exponential phase in the presence of 20 mM H_2_O_2_ compared to cells expressing 6S RNA. Viability of *E. coli* cells was monitored by plating of serially diluted culture aliquots withdrawn at different time points along the growth curves shown in Figure 4a. Representative individual experiments of different sets of strains grown in parallel: (**a**) WT and Δ*ssrS* only; (**b**) WT and Δ*ssrS* in comparison to complementation strain Δ*ssrS+S*; (**c**) growth of the four complementation strains.

Reduced viability of 6S RNA-deficient cells in the presence of 20 mM H_2_O_2_ was also demonstrated by plating assays (Figure 5). A relative decrease in the number of Δ*ssrS* colonies in comparison to the WT became evident 30 min after H_2_O_2_ addition. After 1 h of H_2_O_2_ treatment, where WT cells also showed reduced viability, Δ*ssrS* bacteria gave rise to only very few colonies. After 48 h, WT cells had recovered from oxidative stress, whereas the Δ*ssrS* culture was devoid of any viable cells (Figure 5a). In contrast, complementation strain Δ*ssrS+S* fully restored the WT phenotype 30 min and 3 h post-stress induction (Figure 5b), whereas strain Δ*ssrS+0* yielded even fewer colonies than Δ*ssrS* bacteria 3 h post-induction (Figure 5b,c).

To exclude any influence of unnoticed genome alterations in our previously constructed Δ*ssrS* strain, we also tested three other well-described *E. coli* WT and corresponding 6S RNA knockout strains [26]. These strains (Table S4) showed the same phenotype, namely decreased viability of the 6S RNA knockout strains under oxidative stress conditions (Figure S12). 

### 2.4. Investigation of 6S RNA Expression under Oxidative Stress Conditions

The possibility that 6S RNA levels might change in response to H_2_O_2_ treatment was investigated by Northern blot analysis of the WT strain (see Supplementary Materials, Sections S3 and S4; Tables S2 and S3). However, we found no evidence for significant changes in 6S RNA expression (Figure S13), neither in the presence of 2 mM or 5 mM H_2_O_2_ when monitoring growth for 24 h (corresponding to Figure 1a) nor under more stringent conditions after the addition of 20 mM H_2_O_2_ (corresponding to Figure 4a).

### 2.5. Screening for Oxidative Stress Response Genes Affected by 6S RNA

**Figure 6:**
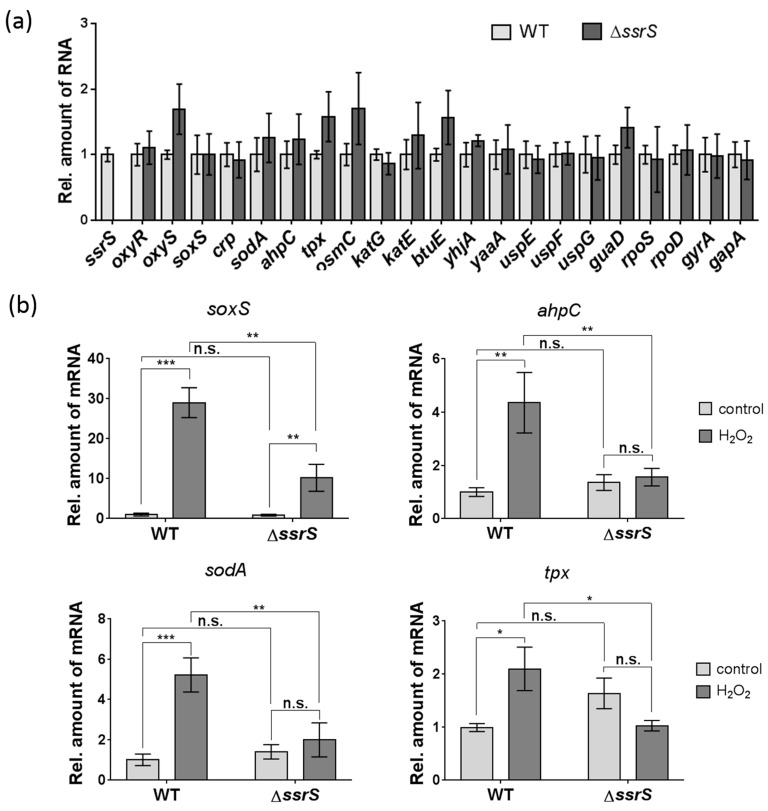
Relative expression levels (qRT-PCR) of selected mRNAs and non-coding RNA OxyS in *E. coli* WT and Δ*ssrS* bacteria. (**a**) Standard conditions (in the absence of H_2_O_2_). No significant changes in expression levels were detected for any of the analyzed genes (standard t-test). (**b**) Genes with dysregulated mRNA levels in *ΔssrS* bacteria under H_2_O_2_ stress. Total RNA samples were isolated either before (control, light grey bars) or 10 min after treatment with 20 mM H_2_O_2_ (dark grey bars). Statistical analysis was performed by the two-way ANOVA test, *p*-values: ***—<0.001; **—<0.01; *—<0.05; n.s.—not significant. Relative amounts of mRNAs were normalized to 16S rRNA and mRNA levels of the WT strain in the absence of H_2_O_2_ were set to “1”.

To identify possible 6S RNA targets that may contribute to reduced viability of the Δ*ssrS* strain, we analyzed the expression levels of a set of genes known to be involved in oxidative stress response by qRT-PCR (Table 1). As a first step, we compared the WT and Δ*ssrS* strain under standard growth conditions. All tested genes showed no significant changes in expression levels upon lack of 6S RNA (Figure 6a). The only difference was a trend toward weak upregulation of genes *oxyS*, *tpx*, *osmC*, *btuE* and *guaD* in the Δ*ssrS* strain. Next, we analyzed expression levels of these genes in the exponential phase of cell growth after exposure to 20 mM H_2_O_2_ (setup as in Figure 4a). To minimize RNA degradation at 20 mM H_2_O_2_ and to preempt progressing lethality of the Δ*ssrS* strain, we withdrew cell aliquots after 10 min of H_2_O_2_ treatment. At this time point, WT and Δ*ssrS* bacteria showed similar numbers of viable colonies (Figure 5a). Total RNAs isolated from both cell cultures before the addition of H_2_O_2_ were used as negative controls. 

The strongest activation upon H_2_O_2_ treatment was demonstrated for the *oxyS* and *katG* genes (~10^2^-fold), however, no difference between the WT and Δ*ssrS* strain was observed (Figure S14). Moderate upregulation under oxidative stress was evident for genes *crp*, *osmC*, *yaaA*, *uspF* and *uspG*. Other genes demonstrated either essentially no sensitivity to H_2_O_2_ treatment (e.g., *katE*, *btuE*, *uspE*, *gapA*) or even downregulation of mRNA levels, e.g., *oxyR*, that is in line with previous observations [35]. However, mRNA levels of four genes among the total setup were revealed to be lower in the Δ*ssrS* versus WT strain under H_2_O_2_ stress (Figure 6b). Namely, expression levels of *soxS* (activator of superoxide response), *ahpC* (alkyl hydroperoxide reductase C), *sodA* (superoxide dismutase) and *tpx* (thiol peroxidase) mRNAs were ~2–3-fold decreased in the absence of 6S RNA. 

### 2.6. Comparative Proteomic Analysis of E.coli 6S RNA Knockout and WT Cells under Oxidative Stress Conditions

As previously performed for *B. subtilis* [19] we further analyzed proteomic differences between the WT and Δ*ssrS* knockout strain by 2D-PAGE (see Supplementary Materials, Section S9, Figure S15), being aware that this method allows only analysis of a fraction of the total cellular proteome. Compared with standard conditions of cell growth (Figure S16a; Table S6), an elevated number of proteins showed significantly changed levels in Δ*ssrS* versus WT bacteria upon treatment with 5 mM H_2_O_2_**,** particularly during exponential growth (Figure S16b; Tables S7 and S8). This included lower amounts of AhpC and Tpx proteins in the Δ*ssrS* strain, in line with the qRT-PCR results for the corresponding mRNAs (Figure 6b)_._

## 3. Discussion

NcRNAs are widely activated in response to different stresses and/or adaptation to unfavorable conditions, where they regulate the expression of single or multiple targets. For example, DsrA, ArcZ, and RprA ncRNAs are known to activate translation of RpoS (σ^38^), the sigma factor specific for stationary phase transcription and stress response [9]. Other small ncRNAs, such as OxyS and RyhB have dozens of mRNA targets, representing genes that are essential for coping with oxidative conditions or iron starvation, respectively [49]. According to transcriptomic data, *E. coli* 6S RNA regulates the expression of hundreds of genes, also including several stress response proteins, translation and transcription factors as well as enzymes involved in various metabolic processes [17,22]. However, such a global influence impedes attempts to identify the most affected targets, for example in analyses of 6S RNA knockout strains where direct and indirect dysregulation effects at multiple levels contribute to the observed physiological state. A first key step in this endeavor is the identification of specific phenotypes of 6S RNA-deficient cells to obtain decisive evidence for certain pathways being affected by this ncRNA. 

Here, we discovered a lethal phenotype of *E. coli ΔssrS* bacteria when exposed to elevated concentrations of H_2_O_2_ (10 to 20 mM). Through complementation using a plasmid-borne *ssrS* gene we were able to fully rescue the lethal phenotype, thus proving that it is a direct consequence of the lack of 6S RNA expression. This is the first reported lethal phenotype of 6S RNA-deficient bacteria in general. So far, phenotypes of 6S RNA knockouts were primarily observed under extreme alkaline or acidic conditions: *E. coli* [23], *B. subtilis* [19,27], *Salmonella* [30]; salt stress: *R. sphaeroides* [28], *B. subtilis* [27]; reduced viability upon exposure to nutrient starvation: *E. coli* [21], *B. subtilis* [19,25,29]; or reduced bacterial pathogenicity [31,32]. Notably, the oxidative stress response is one of the most important defense mechanisms, especially in *E. coli* and related *Enterobacterales*, including pathogenic species [50]. There are a lot of reasons for the endogenous formation of reactive oxygen species (ROS), but a number of oxidative stress factors also originate from the environment [51]. Moreover, the accumulation of ROS either for dietary reasons or due to antibiotic therapy widely impacts human microbiota in the gut [52].

The general defense of *E. coli* against damage by oxidative stress is well-studied, including the key regulators OxyR and SoxRS, the alternative sigma factor RpoS and many other proteins that are conserved among Proteobacteria [36]. OxyR is a transcription factor that is activated through oxidation by H_2_O_2_, which triggers the formation of an intramolecular disulfide bond [53]. Genes that are activated by oxidized OxyR include *katG* (catalase-peroxidase), *dps* (involved in DNA damage repair & iron storage), the *ahpCF* operon (alkyl hydroperoxide reductase, [AhpC]_10_[AhpF]_2_) and *oxyS* (see below) [54,55,56,57]. SoxRS stands for a two-stage regulation system, where SoxR induces expression of SoxS that activates the expression of 20 or more genes. Overall, transcription factors OxyR, SoxR and SoxS were reported to regulate the transcription of 68 genes in 51 transcription units in *E. coli* MG1655 cells [55]. Activation of these stress response regulons leads to increased expression of different H_2_O_2_-degrading enzymes, among them the major catalases KatG and KatE as well as alkyl hydroperoxide reductase AhpCF. Recent studies discovered some novel protein players as well, for example, the transcriptional regulator LsrR that directly inhibits *ahpCF* and *katG* promoters [58]. 

NcRNAs also contribute to the oxidative stress response in bacteria [59]. The first described ncRNA regulator, MicF, for long considered to have only *ompF* mRNA as a target, also affects the regulatory network involving the leucine responsive protein Lrp [60]. The most famous ncRNA in this context is OxyS—a H_2_O_2_-inducible (via OxyR) ncRNA that represses expression of transcription factors, such as NusG, FhlA and RpoS, which entails pleiotropic alterations in the expression of many other genes [59]. Similar to our 6S RNA knockout, deletion of OxyS in *E. coli* was also shown to cause lethality after growth for 20 min at 5 mM H_2_O_2_ [61]. Notably, OxyS is highly activated upon H_2_O_2_ stress but is not transcribed under normal conditions [38]. By contrast, 6S RNA is constitutively expressed, with the highest levels in stationary phase, and is not activated in response to H_2_O_2_ treatment, as shown in the present study (Figure S13). Nevertheless, despite being a global regulator of the transcription machinery, 6S RNA may well impact on transcription of specific genes involved in H_2_O_2_ degradation. To explore this possibility, we analyzed the expression levels of 21 known players in the oxidative stress response by qRT-PCR in the Δ*ssrS* versus WT strain. While no substantial differences between the two strains were observed under standard growth conditions, we indeed saw a ~2 to 3-fold downregulation of *soxS*, *ahpC*, *sodA* and *tpx* mRNA levels upon treatment with H_2_O_2_. As alkyl hydroperoxide reductase AhpC, superoxide dismutase SodA and thiol peroxidase Tpx directly degrade H_2_O_2_ and related ROS [42,62,63], their deficiency very likely contributes to the lethal phenotype of the Δ*ssrS* strain owing to insufficient or slow decomposition of the stress reagent. Finally, the H_2_O_2_–inducible global transcription regulator SoxS targets at least 20 genes [55], including *sodA* and its own gene [63]. It is, therefore, likely that the attenuated H_2_O_2_-induced decrease in the level of *sodA* mRNA (and potentially other mRNAs not yet identified) in the Δ*ssrS* strain has its origin in reduced expression of SoxS. Moreover, *ahpC* is under control of OxyR whose mRNA levels were not significantly affected by the lack of 6S RNA, in line with previous observations that *oxyR* expression is not induced upon H_2_O_2_ stress [35]. Thus, abolished H_2_O_2_ induction of *ahpC* expression in the Δ*ssrS* strain (Figure 6b) is expected to have other reasons than 6S RNA effects on *oxyR* expression.

Finally, we performed a comparative proteomic analysis (based on 2D-PAGE) of the Δ*ssrS* and parental *E. coli* WT strain, which unveiled a number of dysregulated proteins, especially under oxidative stress conditions. Most interesting in the context of this study was, among other proteins, the identification of lower levels of proteins AhpC and Tpx in Δ*ssrS* bacteria (Figure S16, Table S7). This correlates with the trend toward fewer amounts of their mRNAs in Δ*ssrS* versus WT bacteria upon exposure to H_2_O_2_ (Figure 6b), despite the fact that qRT-PCR and proteome analysis were performed in different conditions of cell growth for technical reasons (e.g., 20 versus 5 mM H_2_O_2_, respectively). Interestingly, the dysregulated proteins identified so far in Δ*ssrS* bacteria included AphA (acid phosphatase) and DsbA (thiol:disulfide interchange protein) whose mRNA levels were also found to be altered in microarray analyses of Δ*ssrS* bacteria in standard growth conditions. Levels of *aphA* mRNA were reported to be upregulated (~1.7-fold) and those for *dsbA* mRNA to be downregulated (~1.6-fold) in Δ*ssrS* cells [17]. These data perfectly correlate with our findings that AphA protein levels are increased in Δ*ssrS* cells, while DsbA levels decreased (Table S7). Despite the fact that DsbA is not a primary factor of the oxidative stress response, it is yet a key player in the correct folding of proteins within the periplasmic space of *E. coli* to counteract protein misfolding and aggregation in the presence of elevated ROS levels. Moreover, DsbA is a prospective target for novel antimicrobial agents and antibiotics [64].

Notably, we previously observed that AhpC and Tpx were upregulated in the proteomes of *B. subtilis* strains that lack either 6S-1 or 6S-2 RNA, while SodA was only upregulated in the 6S-2 knockout strain [19]. In the recent study investigating 6S RNA-deficient strains derived from an undomesticated *B. subtilis* strain, ∆6S-1&2 RNA double knockout cells also showed a prolonged lag phase under mild oxidative stress conditions [27]. These findings suggest the involvement of 6S RNAs in the regulation of oxidative stress responses in phylogenetically distant bacterial species.

## 4. Materials and Methods

### 4.1. Bacterial Strains and Plasmids

All plasmids and *E. coli* strains used in this study are listed in Table 2. Construction of *ssrS* complementation and corresponding control strains is detailed in Supplementary Materials (Section S2, Figures S3–S5, Table S1). Cells were usually grown in LB medium (5 g/L NaCl, 10 g/L peptone, 5 g/L yeast extract, pH 7.5) at 37 °C with continuous stirring (200 rpm). All nutrient media were prepared in distilled water and sterilized by autoclaving. Solid LB agar media additionally contained 1.5% (*w/v*) agar. Further *E. coli* control strains (three pairs of WT/Δ*ssrS* strains) are listed in Table S4 and were kindly provided by Karen Wassarman [23].

### 4.2. Growth Curve Measurements in Flasks

For growth curve monitoring, *E. coli* strains were grown in the absence of antibiotic in liquid cultures (100 mL of LB medium) in Erlenmeyer flasks (500 mL volume) covered with a metal cap. In general, freshly autoclaved LB medium was inoculated with a stationary phase culture (grown from a single colony overnight at 37 °C, 200 rpm, in the presence of antibiotic) to a starting OD_600_ of 0.05 or 0.1, followed by growth at 37 °C under stirring (200 rpm) in a waterbath shaker. Culture aliquots were withdrawn at indicated time points and diluted 1:10 in fresh LB medium for measurement of optical density at 600 nm. To plot growth curves, at least three biological replicates of the same growth experiment were conducted. For oxidative stress experiments, appropriate amounts of 1 M H_2_O_2_ prepared from an aqueous 30% (*w/w*) H_2_O_2_ stock (9.8 M; Sigma-Aldrich, St. Louis, MO, USA) and standardized by KMnO_4_ (Sigma-Aldrich) titration were added to LB media prior to inoculation. In another set of experiments (indicated in the text), freshly autoclaved media were first inoculated with a stationary phase culture to a starting OD_600_ of 0.1. After incubation for ~2 h and achieving an OD_600_ of ~0.5, appropriate amounts of H_2_O_2_ (f.c. 10–20 mM) were added to the cell culture, followed by further cultivation with monitoring of optical density.

### 4.3. Growth Curve Measurements in Plates with Manual Monitoring of Optical Density

Appropriate amounts of stationary phase culture (grown overnight at 37 °C, 200 rpm, as mentioned above) were diluted to an OD_600_ of 0.05 or 0.1 in LB medium without antibiotic but beforehand supplemented with H_2_O_2_ (concentrations indicated in the corresponding graphs), and then loaded with a multichannel pipette in 100-µL aliquots into the wells of a 96-well plate (Corning, Corning, NY, USA). In a set of initial experiments (Figure 1b, Figures S1 and S2b), plates were covered by plastic lids and then incubated in a conventional air incubator at 37 °C with continuous stirring at 200 rpm. At time points of interest (in general every hour) plates were manually transferred into a plate reader for measurement of OD_600_. In another set of experiments, plates were incubated (37 °C, 160 rpm) in an automated TECAN Safire 2 Platereader (TECAN trading AG, Männedorf, Switzerland) using flat bottom, transparent 96-well microtiter plates with plastic cover (Greiner BIO-ONE, Frickenhausen, Germany). 

### 4.4. Estimation of Cell Survival on Agar Plates

To ensure that the decrease of optical density corresponds to enhanced cell mortality, we withdrew 100 μL aliquots of cell culture grown in flasks at several time points after the addition of H_2_O_2_. From such aliquots, we made a number of serial 10-fold dilutions in fresh LB medium and plated 5-μL drops on solid agar plates without antibiotic. The number of surviving colonies was evaluated by visual inspection. 

### 4.5. Inhibition Zone Assays

For additional estimation of the sensitivity of *E. coli* strains to oxidative reagents we performed classical inhibition zone assays as described previously [28]. In brief, cells were grown to an OD_600_ of ~0.5, then diluted to an OD_600_ of 0.1 with fresh LB medium, followed by adding a 100-μL aliquot to 6 mL of warm (42 °C) top agar (0.8% *w/v*); the mixture was subsequently poured on top of a solid agar (1.5% *w/v*) plate. A filter paper disk (d = 5 mm) was soaked with a H_2_O_2_ solution of defined concentration and positioned in the center of the rigidified top agar. The diameter of the inhibition zone was measured with a ruler after incubation for 24 h at 37 °C.

### 4.6. Gel Eletrophoresis and Northern Blotting

Expression levels of 6S RNA in the WT and complementation Δ*ssrS+S* and WT+*S* *E. coli* strains were estimated by Northern blot analysis (both in standard conditions and in the presence of 2 mM, 5 mM or 20 mM H_2_O_2_) following the protocols described in [66,67]. For more details see Supplementary Materials, Sections S3 and S4.

### 4.7. Reverse Transcription and qRT-PCR

Total RNA samples (0.5 μg) isolated from *E. coli* by TRIzol reagent (Thermo Fisher Scientific, Waltham, MA, USA) were treated with DNase I (Thermo Fisher Scientific, Waltham, MA, USA) followed by reverse transcription using the OT-1 Kit with MMLV Reverse Transcriptase (Syntol, Moscow, Russia) according to the manufacturer’s protocol (reaction volume: 20 µL). Thereafter, the volume was increased to 40 μL with ddH_2_O and 2 μL of such diluted cDNA samples were mixed with 4 μL 5× qPCR mix-HS SYBR (Evrogen, Moscow, Russia) and 0.4 μM (f.c.) of each reverse and forward primer (Table S5) in a final volume of 20 μL. Three-step cycling reactions were conducted using a CFX Connect Real-Time PCR Detection System (Bio-Rad Laboratories Inc., Hercules, CA, USA). The amount of RNAs was calculated from threshold cycles (C_t_) by the 2^−∆∆Ct^ method using the amount of 16S rRNA for normalization. At least three biological replicates were measured for each sample and used to calculate mean 2^−∆∆Ct^ values. 

### 4.8. Comparative Proteome Analysis

For this experiment, *E. coli* MG1655 WT and Δ*ssrS* knockout cells were grown in parallel in 100 mL LB medium starting from an OD_600_ of 0.1 (inoculated from overnight cultures, either in the absence or presence of 5 mM H_2_O_2_). 50 mL aliquots were withdrawn during exponential growth at OD_600_ ~ 1; the rest of the cell culture was withdrawn in stationary phase after further incubation for up to 12 h. After centrifugation at 4000 rpm (10 min, 4 °C), cell pellets were snap-frozen in liquid nitrogen and stored at −80 °C. All procedures of sample preparation, labeling and 2D protein gel electrophoresis were carried out exactly as was described for the comparative proteome analysis of 6S RNA-deficient *B. subtilis* strains [19]; for details, see Supplementary Materials, Section S9. After 2D-PAGE, separated fluorescently labeled protein spots were visualized (Figure S16) using a Typhoon FLA 9500 Biomolecular Imager (GE Healthcare, Chicago, IL, USA). After standard fixation (40% (*v*/*v*) ethanol, 10% (*v*/*v*) acetic acid) and silver staining of gels, protein spots of interest were excised, washed and subjected to tryptic digestion and identification of resulting peptides by MALDI-TOF mass spectrometry analysis. Proteins were identified using the Mascot software release version 2.4.2 (Matrix Science) in conjunction with the NCBIprot database.

## Figures and Tables

**Table 1:** Stress response genes selected for qRT-PCR screening.

Gene	Name	Function	References
Major regulators of oxidative stress response			
*oxyR*	H_2_O_2_-inducible genes activator	activator of H_2_O_2_-inducible genes (including *katG*, *ahpC*, *oxyS*)	[36,37]
*oxyS*	non-coding RNA OxyS	regulates expression of a number of genes by interaction with mRNAs via antisense mechanism	[38]
*soxS*	regulatory protein SoxS	RNAP-binding protein, activator of superoxide response	[37]
*crp*	cAMP-activated global transcriptional regulator	activates transcription by RNAP recruitment	[39]
Proteins involved in degradation of H_2_O_2_ and/or other ROS *			
*katG*	catalase-peroxidase	degradation of H_2_O_2_	[37]
*katE*	catalase HPII	degradation of H_2_O_2_	[37]
*yhjA*	cytochrome c peroxidase Ccp	degradation of H_2_O_2_	[40]
*btuE*	thioredoxin/glutathione peroxidase BtuE	non-specific peroxidase, degradation of H_2_O_2_	[41]
*ahpC*	alkyl hydroperoxide reductase subunit C	degradation of H_2_O_2_ and organic hydroperoxides	[37]
*tpx*	thiol peroxidase	degradation of H_2_O_2_ and organic hydroperoxides	[42]
*sodA*	superoxide dismutase [Mn]	degradation of superoxide anion radicals	[43]
*osmC*	peroxiredoxin OsmC	degradation of organic hydroperoxides	[44]
General stress proteins			
*rpoS*	RNA polymerase sigma factor RpoS	stationary phase and general stress response gene activation	[45]
*yaaA*	peroxide stress resistance protein YaaA	protects DNA from oxidative damage	[46]
*uspE*	universal stress protein E	general response to different environmental stresses including anti-oxidative function, essential for cellular adhesion, agglutination, cell motility and swimming	[47]
*uspF*	universal stress protein F		
*uspG*	universal stress protein UP12		
Control proteins			[48]
*rpoD*	RNA polymerase sigma factor RpoD, σ^70^	primary sigma factor during exponential growth	
*gyrA*	DNA gyrase subunit A	type II topoisomerase, DNA supercoiling	
*guaD*	guanine deaminase	guanine degradation	
*gapA*	glyceraldehyde-3-phosphate dehydrogenase A	glycolysis	

* Reactive Oxygen Species.

**Table 2:** Strains and plasmids used in this study.

Strain or Plasmid	Genotype ^1^	Reference or Source
p177_*rnpB*	pACYC177 *rnpB amp* (Amp^r^)	[65]
p177_*ssrS*	pACYC177 *ssrS amp* (Amp^r^)	This work
p177*_empty*	pACYC177 (Amp^r^) (Kan^r^)	Lab stock
WT (MG1655)	*E. coli* K-12 MG1655 F- λ- *ilvG*- *rfb*-50 *rph*-1	Lab stock
Δ*ssrS*	MG1655 *ssrS*::*kan* (Kan^r^)	[34]
Δ*ssrS+S*	MG1655 *ssrS*::*kan* (Kan^r^) + *p177_ssrS*(Amp^r^)	This work
WT*+S*	MG1655 + *p177_ssrS* (Amp^r^)	This work
Δ*ssrS+0*	MG1655 *ssrS*::*kan* (Kan^r^) + *p177_empty* (Amp^r^)	This work
WT*+0*	MG1655 + *p177_ empty* (Amp^r^)	This work

^1^ Kan^r^, kanamycin resistance; Amp^r^, ampicillin resistance. The final antibiotic concentrations in *E. coli* growth cultures were 100 mg/mL ampicillin, 10 mg/mL kanamycin.

## Supplementary Materials

The following supporting information can be downloaded at: https://www.mdpi.com/article/10.3390/ijms23073653/s1.
