# Supplementary material for: Involvement of E. coli 6S RNA in Oxidative Stress Response
Source: Int J Mol Sci. 2022 Mar 26;23(7):3653. doi: 10.3390/ijms23073653 (PMC8998176; doi:10.3390/ijms23073653)
Supplement: Supplementary file 1 [file ijms-23-03653-s001.zip › ijms-1638637-supplementary.pdf]

Supplementary Materials

1. Cultivation of MG1655 (WT) and Δ*ssrS* strains under oxidative stress conditions

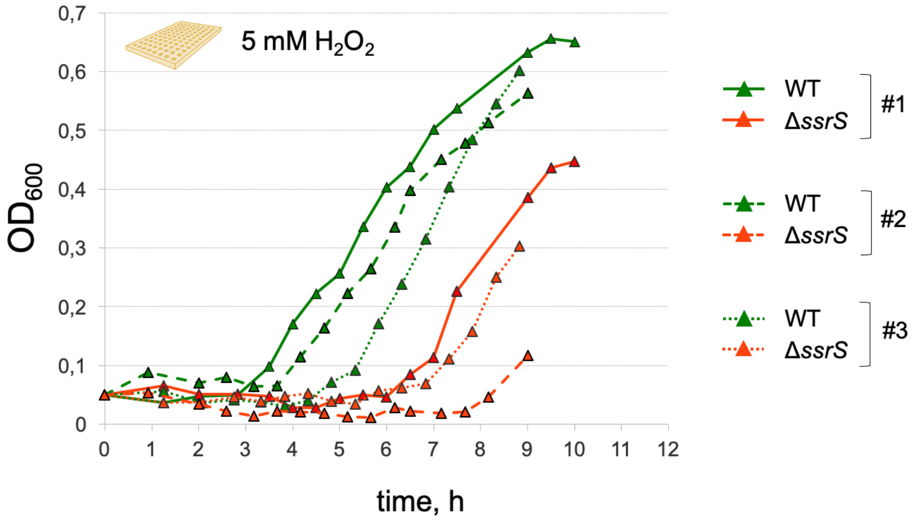

Figure S1. Lack of 6S RNA leads to a delay (2, 4 or 5 h) in outgrowth of cell cultures grown in the presence of 5 mM H_2O_2 in 96-well plates with manual monitoring of optical density at 600 nm. Independent growth curves (solid, dashed or dotted lines) corresponding to individual experiments conducted on different days and representing cultures inoculated with colonies derived from independent agar plates.

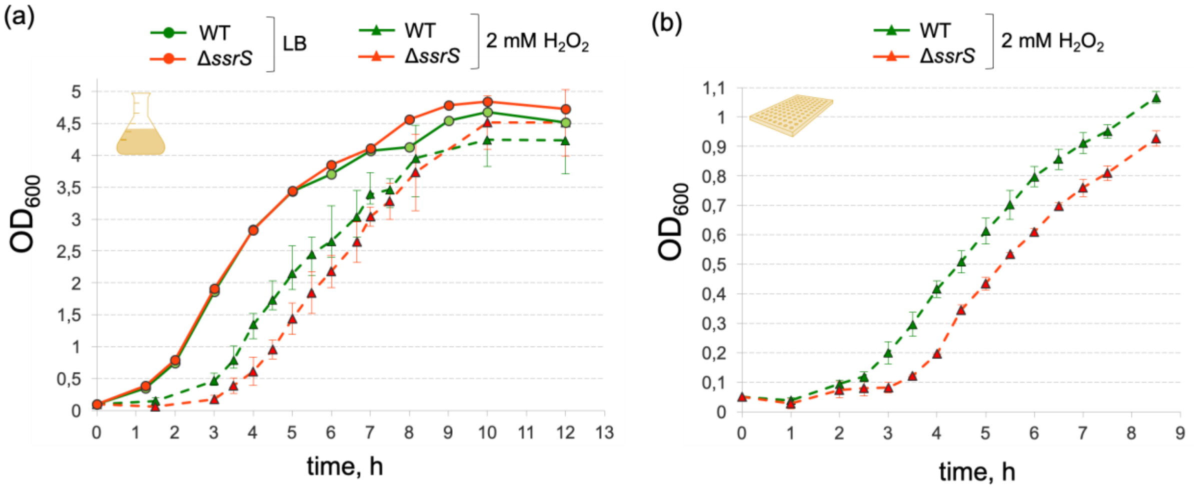

Figure S2. Lack of 6S RNA leads to a delay in outgrowth of cell cultures grown in the presence of 2 mM H_2O_2. Cultivation in flasks (**a**) or in 96-well plates (**b**) with manual monitoring of optical density at 600 nm. For each experiment in the presence of 2 mM H_2O_2 in panels a and b, three biological replicates were grown in parallel; only single replicates were grown in case of the LB control cultures in panel a.

2. Construction of complementation strains

Since we aimed at complementation of the *ssrS* gene without significantly overexpressing 6S RNA, we used the low copy plasmid pACYC177 with a p15A origin, specifically the pACYC177 derivative p177_*rnpB* (3533 bp) from our lab stock [1], which harbors the *E. coli rnpB* gene (encoding RNase P RNA) with its native promoter and transcription terminator; in addition, p177_*rnpB* encodes a functional ampicillin (Amp) resistance gene (Figure S3). First, using *E. coli* MG1655 genomic DNA as a template we amplified the *E. coli* 6S RNA gene including 96 bp of upstream sequence (harboring the P1 promoter), simultaneously introducing a cleavage site for the restriction endonuclease NheI at the 5'-end. For this purpose, we used primers cl1_Ec6S_Fw and cl1_Ec6S_Rv (Table S1). The resulting 282-bp PCR fragment was purified by agarose gel electrophoresis. Second, we amplified a region

encoding the transcription terminator sequence only (without amplification of the RNase P RNA coding region) using primers cl2_rnpB_Fw and cl2_rpnB_Rv (Table S1); this introduced a BamHI restriction site at the 3′-end of the terminator sequence and, at the 5'-end, a 20 nt long sequence complementary to the 3'-end of the *ssrS* gene. Subsequent overlap PCR using the two PCR products of the previous steps resulted in a DNA fragment of 1186 bp that was purified and cloned into p177_*rnpB* via NheI/BamHI restriction sites. The final p177_*ssrS* vector (Figure S4) encoded mature 6S RNA under control of its native P1 promoter with a strong Rho-independent transcription terminator directly at the end of the *ssrS* gene (Figure S5). Plasmid p177_*ssrS* was initially transformed into chemically competent *E. coli* DH5α cells and clones were selected on agar plates containing 100 µg/µL Amp, followed by plasmid purification and confirmation of appropriate insertions/deletions by restriction analysis. As a negative control we used the parental pACYC177 (3941 bp) vector that we named p177_ *empty* for convenience. Finally, p177_*ssrS* and p177_*empty* were transformed into chemically competent *E. coli* MG1655 WT and Δ*ssrS* cells (resulting in four new strains, abbreviated as WT+*S*, Δ*ssrS*+*S*, WT+*0*, Δ*ssrS*+*0*); plasmids were purified from each strain and confirmed by DNA sequencing.

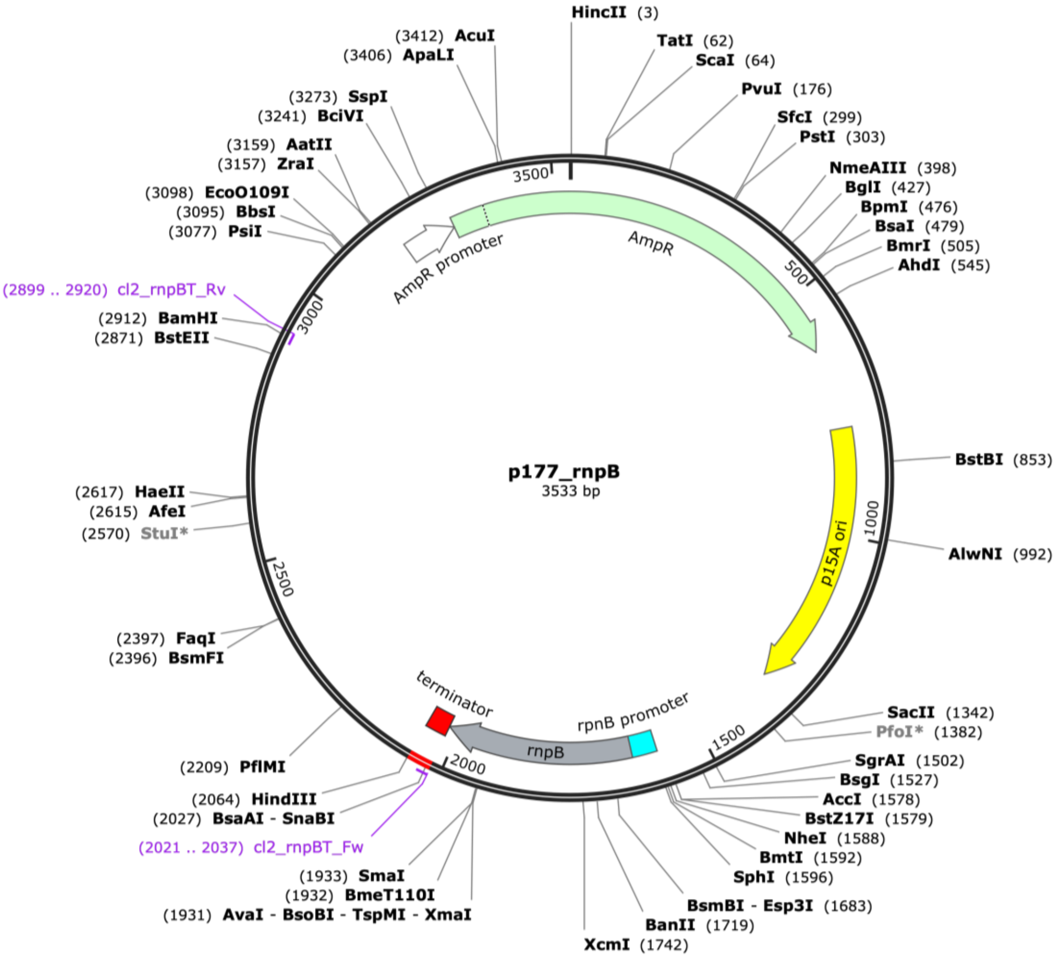

Figure S3. Plasmid map of p177_*rnpB*.

Table S1. Primers used for cloning.

Name	Sequence*, 5'–3'
cl1_Ec6S_Fw	<u>*CAGGCT*</u>AGCGTTACTTGAACAAGGTCGC
cl1_Ec6S_Rv	GGAATCTCCGAGATGCCGC
cl2_rnpB_Fw	<u>*GGCGGCATCTCGGAGATTCC*</u>GATTTACGTAAAAACCC
cl2_rnpB_Rv	CTAGGATCCTCCGGCGTTCAGC

*Overhanging sequences are underlined.

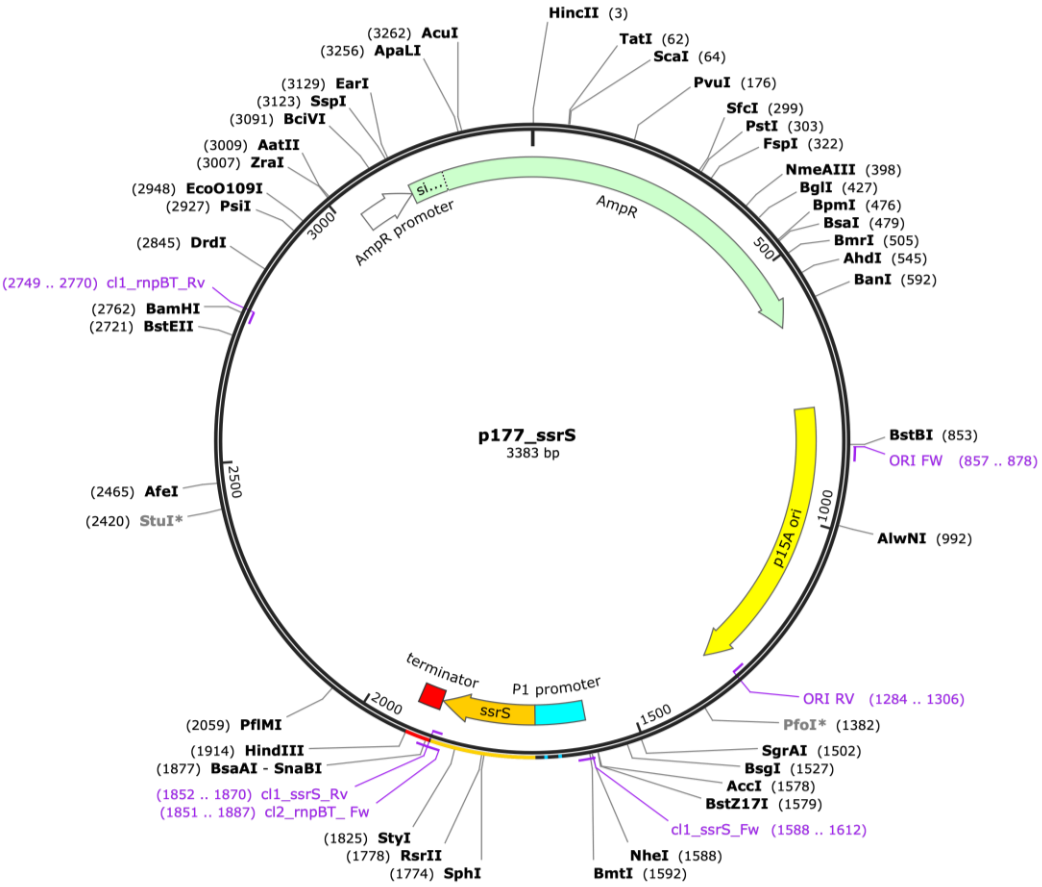

Figure S4. Plasmid map of p177_*ssrS*.

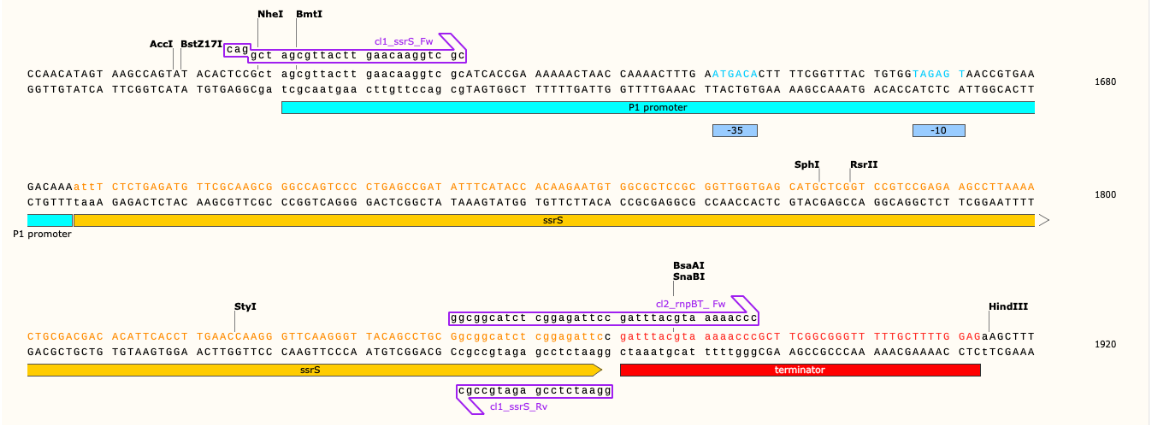

Figure S5. 6S RNA gene region in plasmid p177_*ssrS*.

3. Northern blotting

3.1. *Synthesis of E. coli 6S RNA and probes for Northern blotting*

As hybridization probes, antisense transcripts covering the respective full-length 6S or 5S RNA, internally labeled with digoxigenin-UTP, were synthesized from PCR templates by T7 RNA polymerase according to the DIG RNA Labeling Mix protocol (Roche Diagnostics). PCR DNA templates for T7 transcription, amplified beforehand with the primers listed in Table S2 using genomic DNA of *E. coli* MG1655 cells as template, were purified by agarose gel after electrophoresis and gel elution using the DNA Cleanup Kit (Evrogen, Russia); extracted DNA fragments were concentrated by ethanol precipitation in the presence of 0.1 M NaOAc, pH 5. *In vitro* transcriptions were set up according to the manufacturer's protocol (Roche Diagnostics) in a final volume of 20 µl. Then 10 µl of each T7 reaction mixture (containing DIG-labeled transcripts) were heated up to 95°C for 5 min, followed by direct addition to 10 mL of hybridization buffer (Hyb Easy Granules, Roche Diagnostics) for Northern blotting.

As positive control for Northern blotting we used *E. coli* 6S RNA that was transcribed from a PCR template (primers 6S_T7_F and 6S_R, Table S2) with T7 RNA polymerase as described in [2] using the HiScribe T7 High Yield RNA Synthesis Kit (New England Biolabs) according to the manufacturer's protocol. After preparative 7.5% denaturing (7 M urea) PAGE, 6S RNA was extracted from the gel followed by ethanol precipitation. The RNA pellet was redissolved in ddH_2O and the RNA concentration was determined by UV spectroscopy.

As radioactive probes we used synthetic DNA oligonucleotides (Table S3) complementary to *E. coli* 6S and 5S RNAs that were 5′-endlabeled by ^{32}P according to standard protocols using T4 polynucleotide kinase (Thermo Fisher Scientific) and [γ-^{32}P]ATP. For membrane hybridization, ~ 10 pmol (~1 x 10^6 Cherenkov cpm) of the denatured (95°C, 5 min) oligonucleotide were used.

Table S2. Primers for T7 transcription template production.

Name	Sequence*, 5'–3'
probe6S_T7_F	<u>TAATACGACTCACTATAG</u>GAATCTCCGAGATGCCGCC
probe6S_R	ATTTCTCTGAGATGTTCGCAAGCG
probe5S_T7_F	<u>TAATACGACTCACTATAGG</u>ATGCCTGGCAGTTCCCTACTC
probe5S_R	TGCCTGGCGGCAGTAG
6S_T7_F	<u>TAATACGACTCACTATAG</u>GATTTCTCTGAGATGTTCGCAAGC
6S_R	GAATCTCCGAGATGCCGC

*Sequence of the T7 promoter is underlined.

Table S3. Sequence of DNA probes specific for 6S and 5S RNA used for radioactive Northern blot detection.

Name	Sequence, 5'–3'
probe6S	CATGCTCACCAACCGCGGAGCGCCACATTC
probe5S	GCGCTACGGCGTTTCACTTCTGAGTTCGGCAT

3.2. *Gel electrophoresis and Northern blotting*

The following procedures were conducted as described [2,3]. In brief, aqueous total RNA (10 µg/sample) in a volume of 10 µL was mixed with an equal volume of denaturing gel loading buffer [0.02% (w/v) bromophenol blue, 0.02% (w/v) xylene cyanol blue, 2.6 M urea, 66% (v/v) formamide, 2 x TBE] and heated up to 95°C for 5 min followed by snap cooling on ice and further loading onto a denaturing 8% polyacrylamide (PAA) gel containing 7 M urea. After gel electrophoresis in 1 x TBE running buffer, RNA samples were transferred onto a positively charged nylon membrane (Roche Diagnostics, Cat. No. 11209299001) using a semi-dry blotter (Helicon, Russia) with 75 mA/cm^2 and 0.5 x TBE as transfer buffer, followed by incubation for 1 h at 80°C to immobilize the RNA on the membrane. Before hybridization, the membrane was preincubated (42°C, 2 h) in 10 mL hybridization buffer (Hyb Easy Granules solution, Roche Diagnostics); then, the prehybrdization solution was replaced with a fresh 10-mL aliqout of the same buffer containing the respective hybridization probe, followed by overnight incubation (68°C). In case of DIG-labeled probes we further followed the manufacturer's instructions (DIG Wash and Block Buffer Set & DIG Detection Set, Roche Diagnostics). Radioactive membranes were washed twice with wash buffer [0.75 M NaCl, 75 mM sodium citrate, 0.01% (w/v) SDS, pH 7.0] and signals were visualized on a Typhoon FLA 9500 Biomolecular Imager scanner (GE Healthcare). To calculate the data, the Quantity One program (Bio-Rad Laboratories) was used. The signal intensity of 6S RNA was normalized to the corresponding 5S RNA signal.

3.3. *Extraction of total RNA*

Cell samples (10–50 mL) were withdrawn from growing cell cultures at the time point of interest and harvested by centrifugation (12,000 x g, 4°C). For subsequent qRT-PCR experiments, total RNA was isolated using the TRIzol reagent (Thermo Fisher Scientific) as described by the manufacturer. For Northern blot analysis, total RNA was isolated by the hot phenol method followed by precipitation with sodium acetate and ethanol as described [3]. The quality of extracted total RNA ewas checked by denaturing (7 M urea) PAGE and RNA concentrations were measured by UV spectroscopy.

4. Confirmation of 6S RNA expression in complementation strains

To confirm successful complementation, we checked expression levels of 6S RNA in each strain by Northern blotting (Figure S6a). Evidently, in the exponential phase of cell growth, 6S RNA is expressed at 2 to 3-fold higher levels in plasmid-complemented WT+*S* and Δ*ssrS*+*S* bacteria compared with the WT (Figure S6a, b). However, in stationary phase, 6S RNA amounts in the complementation strains were comparable (less than 1.2-fold higher) relative to those in the WT or WT+*0* strains. The slight increase of 6S RNA levels in strains carrying plasmid p177_*ssrS* likely reflects a gene dosage effect. Nonetheless, complementation with the *ssrS* gene expressed from a low-copy plasmid avoided substantial overexpression of 6S RNA. Evidently, 5'-end processing of 6S RNA is a

universal feature of all studied strains, as we detected a weak upper band (precursor) in all cases (mainly in exponential and transition phase of cell growth; Figure S6a). The upper band is not present in the case of mature 6S RNA (positive control) obtained by T7 transcription.

The presence of plasmid did not influence the growth behavior in standard LB medium in comparison to MG1655 WT and Δ*ssrS* cells (Figure S7).

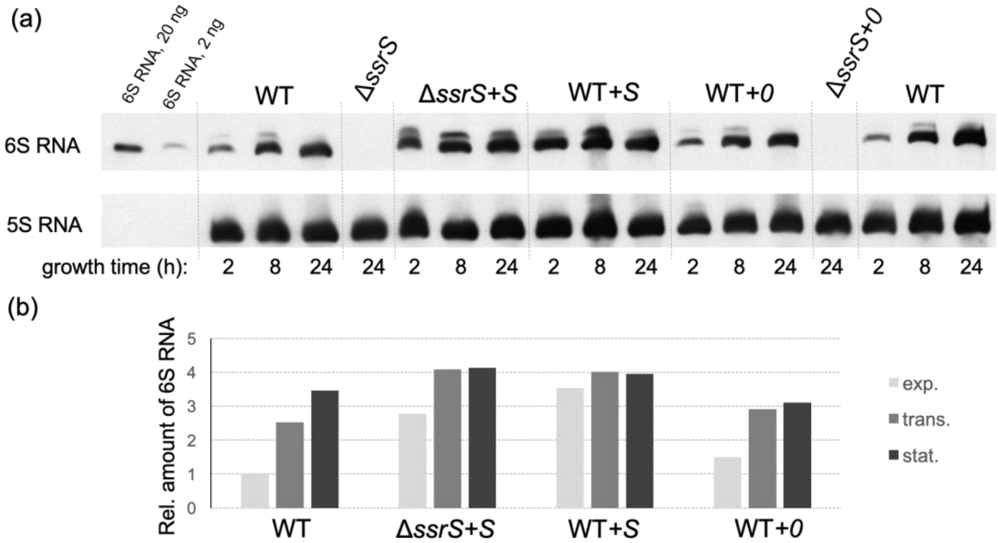

Figure S6. Expression of 6S RNA in MG1655 WT, Δ*ssrS* and derived complementation strains. (**a**) Northern blotting of total RNA extracts (10 μg) obtained from complementation strains (grown in standard LB medium) in comparison to the MG1655 WT (two replicates) with DIG-labeled probes against *E. coli* 6S RNA or 5S RNA. RNA extracts from Δ*ssrS* and Δ*ssrS+0* bacteria were used as negative controls. Cells were withdrawn for total RNA extraction after 2 h (exponential phase), 8 h (transition phase) or 24 h (stationary phase). Pure 6S RNA (20 ng and 2 ng) obtained by T7 transcription was used as positive control. (**b**) Relative abundance of 6S RNA after normalization to 5S RNA. For the WT strain average amounts of two replicates are shown and the value in the exponential phase was set to "1".

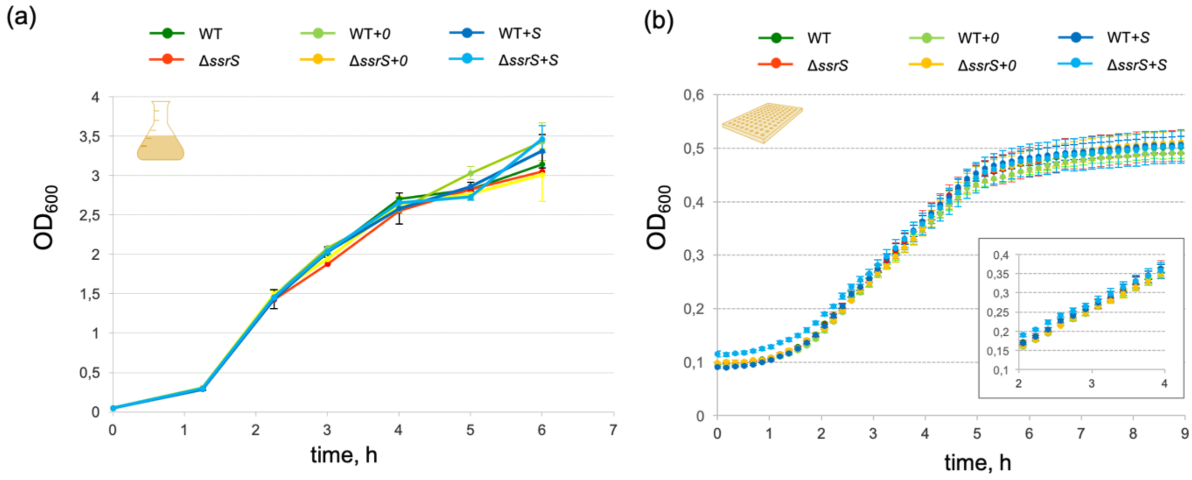

Figure S7. Growth curves of complementation strains (harboring either plasmid p177_*ssrS* or p177_ *empty*) in comparison to the parental MG1655 WT and Δ*ssrS* (knockout) strains (**a**) in standard LB medium (cultivation in flasks) with manual monitoring of optical density at 600 nm or (**b**) in 96-well plates monitored by an automated scanning reader. Representative experiments, in each case with 3 biological replicates grown in parallel.

5. Investigation of knockout phenotype under oxidative conditions

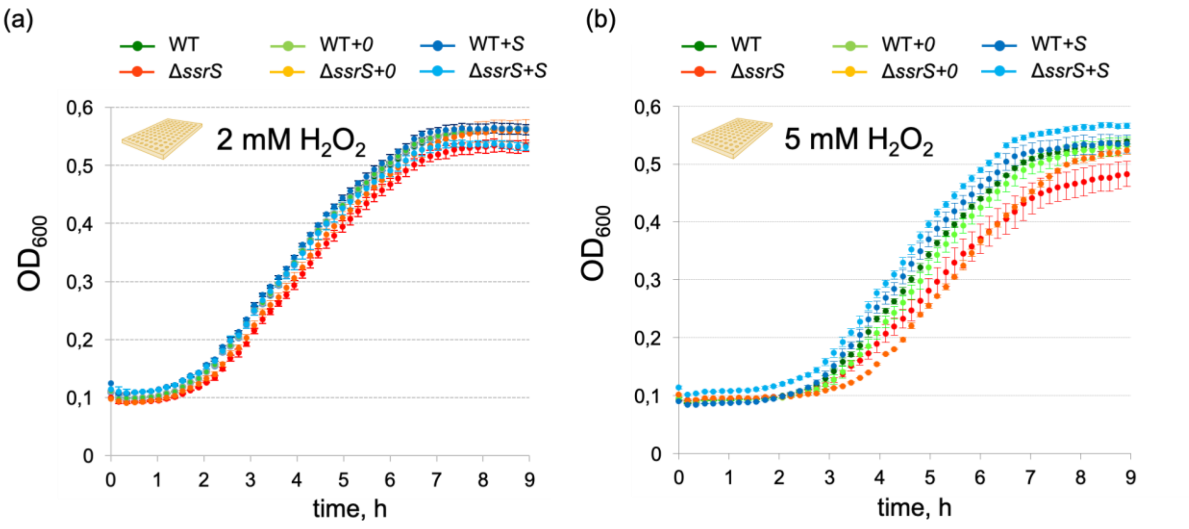

Figure S8. Growth curves of *E. coli* strains in the presence of 2 mM (**a**) or 5 mM (**b**) H_2O_2 during cultivation in 96-well plates monitored by an automated scanning reader; in this setup, the H_2O_2-supplemented LB medium was inoculated from a stationary phase overnight culture that had been grown in LB without H_2O_2.

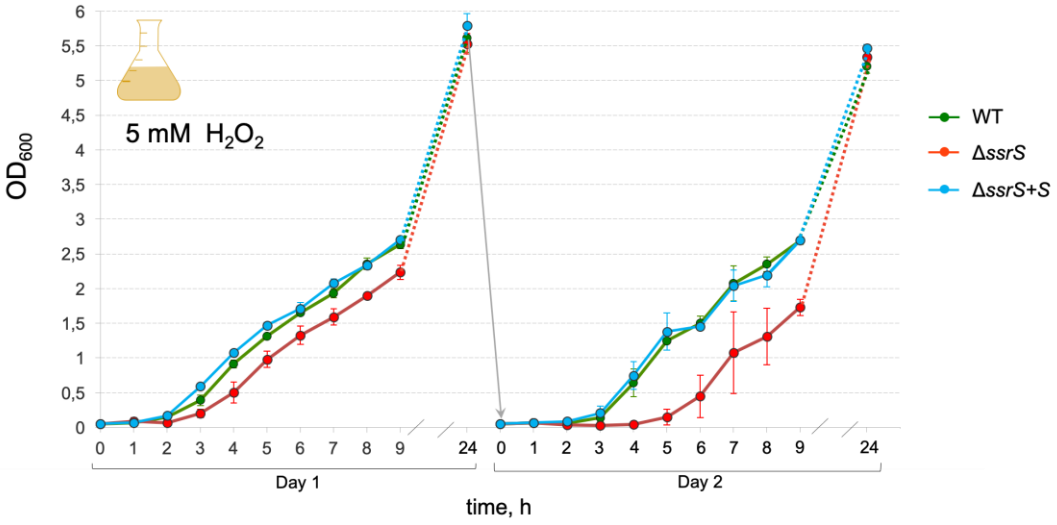

Figure S9. Reproducibility of the Δ*ssrS* phenotype in consecutive growth experiments in the presence of 5 mM H_2O_2 during cultivation in flasks. On day 1, fresh media containing 5 mM H_2O_2 were inoculated (starting OD_{600} = 0.05) with *E. coli* overnight cultures (3 replicates per strain) that had been grown in the absence of H_2O_2. After 24 h, these cultures were used to inoculate fresh media containing 5 mM H_2O_2 (starting OD_{600} = 0.05) to start the second round of growth.

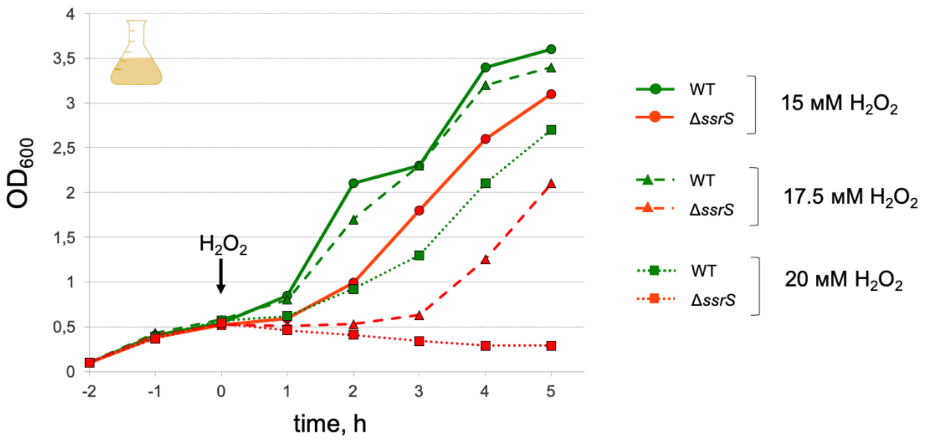

Figure S10. Addition of 15, 17.5. or 20 mM H_2O_2 to exponentially growing *E. coli* Δ*ssrS* cultures incrementally impaired growth and cell survival. Representative growth curves are shown, cultivation was performed in flasks with manual monitoring of optical density at 600 nm.

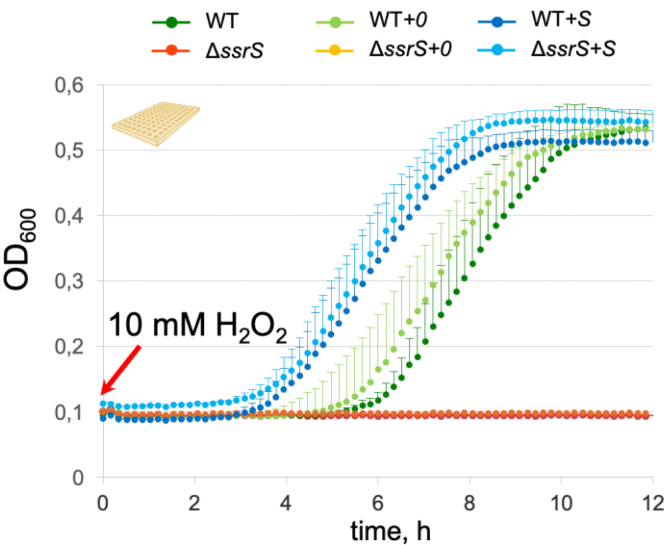

Figure S11. Lack of 6S RNA leads to lethality of *E. coli* cell cultures in the presence of 10 mM H_2O_2 in the plate reader setup. Growth curves of *E. coli* strains in 96-well plates monitored by an automated scanning reader; in this setup, an exponentially growing cell culture (OD_{600} ~ 0.5) was diluted 5-fold in fresh medium containing 10 mM H_2O_2. Three biological replicates of each strain were grown in parallel, enlarged error bars in case of WT+*0* and WT resulted from variation in the exact time of growth delay between individual replicates.

6. H_2O_2 sensitivity of *E. coli* Δ*ssrS* bacteria with other strain backgrounds

Table S4. *E. coli* Δ*ssrS* strains with different strain backgrounds.

Strain	Genotype [1]
E. coli ZK	ZK126 §
E. coli ZKΔ*ssrS*	KW437 (Amp^r) §
E. coli KW	KW72 §
E. coli KWΔ*ssrS*	GS075 (Amp^r) §
E. coli RL	RLG3499 §
E. coli RLΔ*ssrS*	KW348 (Amp^r) §

[1]Amp^r, ampicillin resistance. The final antibiotic concentrations applied to *E. coli* were 100 mg/mL.

§ These are the original *E. coli* strain designations

All strains were kindly provided by Karen Wassarman. For genotypes and details of their construction see ref. [4].

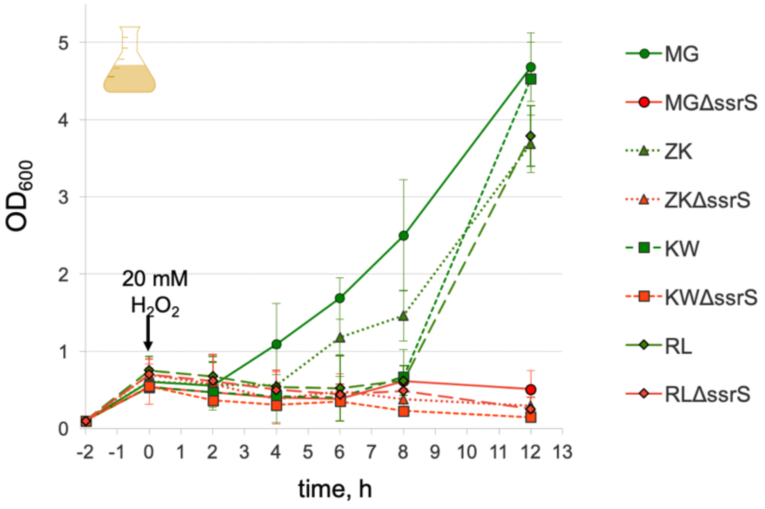

Figure S12. Lethal phenotype of 6S RNA knockouts in different *E. coli* strain backgrounds at 20 mM H_2O_2; setup as in Figure 4c of the main manuscript; for details on strains, see Table S4.

7. Investigation of 6S RNA expression under oxidative stress conditions

Considering that 6S RNA has turned out to be important for *E. coli* survival under oxidative stress conditions, we checked whether its expression is altered in response to H_2O_2 stress. For this purpose, we withdrew aliquots at different time points from flask cultures containing 2 or 5 mM H_2O_2, applying the setup illustrated in Figure 1a of the main text (inoculation from overnight cultures). Extracted total RNA fractions were analyzed by Northern blotting with probes against 6S and 5S RNA. As shown in Figure S13, expression levels of 6S RNA were quite low during exponential phase and accumulated toward stationary phase, regardless of the presence of H_2O_2 in the medium (Figure S13a). RNAs isolated from cultures grown in the presence of 5 mM H_2O_2 were generally of lower quality. We also analyzed 6S RNA levels using the setup where we added 20 mM H_2O_2 to early exponential flask cultures (Figure 4a of the main text). In this case we withdrew samples within 1 h after addition of H_2O_2 from cultures of the WT and Δ*ssrS*+*S* strain. However, no significant changes in 6S RNA levels in response to H_2O_2 treatment were detected for both strains within this 1 h window (Figure 13b). As expected, absolute concentrations of 6S RNA were ~2.5-times higher in the Δ*ssrS*+*S* versus WT strain.

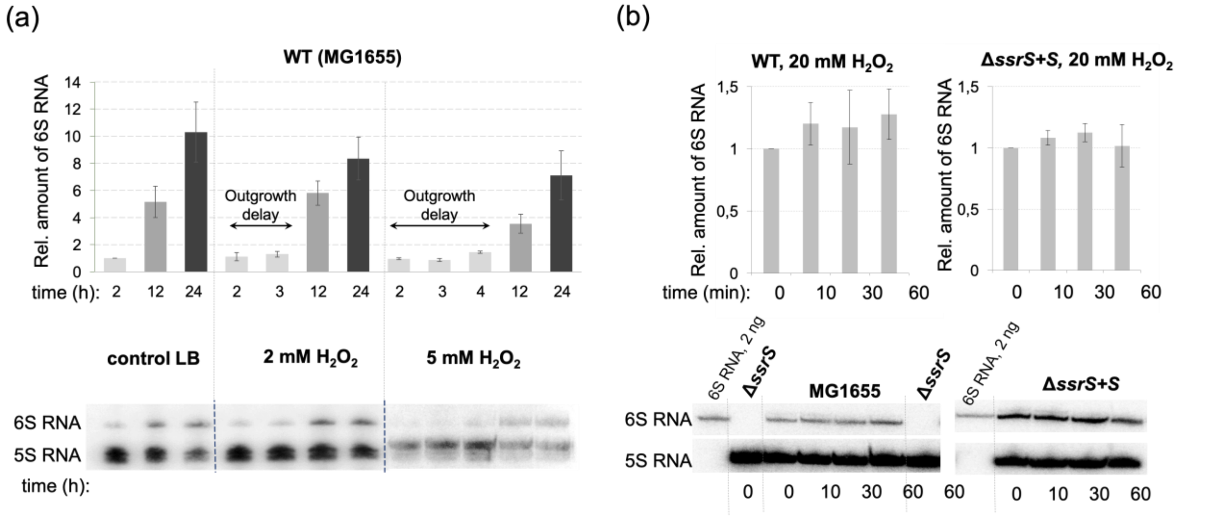

Figure S13. Oxidative stress does not impact *E. coli* 6S RNA expression levels. (**a**) Northern blot analysis of 6S RNA levels (normalized to 5S RNA) in the *E. coli* WT strain during cultivation in the absence (control LB) or presence of either 2 or 5 mM H_2O_2, 3 biological replicates for each condition. 6S RNA signal intensities at the time point 2 h under control LB conditions were set to 1. (**b**) Northern blot analysis (based on 3 biological replicates) of 6S RNA levels in MG1655 WT and Δ*ssrS*+*S* bacteria within 1 h after addition of 20 mM H_2O_2 to early exponential cell cultures. 6S RNA signal intensities at time point 0 h (just before addition of H_2O_2) were set to "1".

8. Dysregulation of mRNA expression in 6S RNA-deficient cells analyzed by qRT-PCR

Table S5. Primers used for qRT-PCR.

Name	Sequence*, 5'–3'	Product length, bp
rrsG_F	GTAATACGGAGGGTGCAAGC	173
rrsG_R	TACGCATTTCACCGCTACAC	
oxyR_F	GGACCAGCCGTAAAGTGTTG	170
oxyR_R	TAGCAGGTACGGTCCAACTG	
yaaA_F	CTCGCTTTCATGACTGGCAG	194
yaaA_R	AGGCTGCATTAAATCGAGCG	
ahpC_F	GTGACGTTGCTGACCACTAC	198
ahpC_R	CCAGACCTTCATCTTCACGC	
soxS_F	ACCAGCCGCTTAACATTGATG	199
soxS_R	TGCGAGACATAACCCAGGT	
guaD_F	GGGCCTATGGTGAGCAATTG	170
guaD_R	AGATTGCGGATGAACAGTGC	
uspF_F	TCACTGCCCTACTATGCCTC	194
uspF_R	GGGATCTTCTTCGCCAATTCC	
katE_F	GATGCGCATAAATTCCCCGA	194
katE_R	ATACCGAAGCCTTCCATGGT	
sodA_F	CACCACACCAAACACCATCA	188
sodA_R	AGACCTTTCCAGAACAGGCT	
gapA_F	ATATGCTGGCCAGGACATCG	165
gapA_R	TCTTTGTGAGACGGGCCATC	
uspG_F	TCACGTACTACCCGGGTCAGC	185
uspG_R	CATTGACTTCATCCCGCACG	
btuE_F	GAACCGGGCAGCGATGAAGAG	171
btuE_R	GGCATAGAATCCGCTCTCTTCC	
yhjA_F	ATACTGCTCCGGAACATCGC	200
yhjA_R	GCCACCAACACCAATCGATG	
uspE_F	ATCTCAATGCTGGCGTTCCC	205
uspE_R	TCTTTCACCATCCACACCGG	
osmC_F	CCTGAAGAACTGATTGGCGC	178
osmC_R	GCACCGCAACTTCACTCTTC	
katG_F	TGCGGATTCTGGTCTGTCTG	174
katG_R	TTCTCCAGAACAGGCAGAGC	
tpx_F	GGCAACTGAGATCGACAACAC	182
tpx_R	CTGCCAGACCTTTCAGTGGG	
ssrS_F	TCCCCTGAGCCGATATTTCATAC	97
ssrS_R	TGTGTCGTCGCAGTTTTAAGG	
rpoS_F	TCCGCTTCTCAACATACGCA	97
rpoS_R	ACGATGTGAATCGGCAAACG	
rpoD_F	GCCGAAGCAGTTTGACTACC	106
rpoD_R	CATTTTGCACTGCTCAACGC	
oxyS_F	GCGGCACCTCTTTTAACCC	95
oxyS_R	CCTGGAGATCCGCAAAAGTTC	

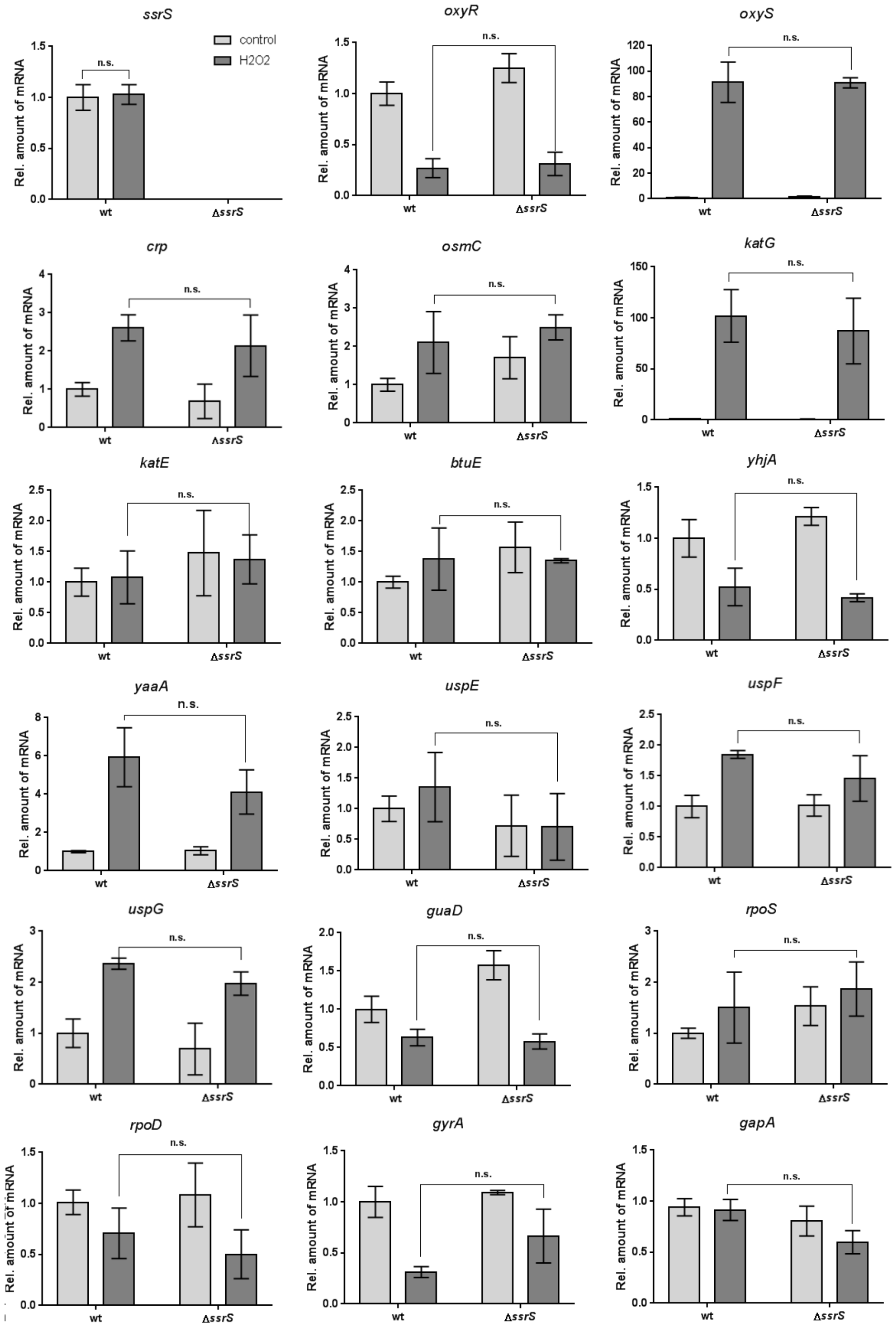

Figure S14. Relative expression levels (qRT-PCR) of selected mRNAs and non-coding RNA OxyS in *E. coli* MG1655 WT and Δ*ssrS* strains. Total RNA samples were isolated either before (control, light gray bars) or 10 min after treatment with 20 mM H_2O_2 (dark gray bars). Statistical analysis was performed by the two-way ANOVA test, n.s. – not significant p-value. Relative amounts of mRNAs were normalized to 16S rRNA and mRNA levels in the MG1655 WT strain grown in the absence of H_2O_2 were set to "1".

9. Comparative proteome analyses

For comparative proteome analysis (see workflow in Figure S15), we grew MG1655 WT and *ΔssrS* bacteria in parallel either in the presence of 5 mM H_2O_2 (corresponds to the growth curves in Figure 1a of the main text) or absence (control), and withdrew aliquots at two time points: in the exponential phase (OD_{600}~0.5) and after 24 h of cultivation (stationary phase, OD_{600}~4). For each condition, we analyzed 2-6 replicates and proceeded with protein identification by MALDI-TOF spectrometry if protein spots were clearly visible in at least two independent experiments.

First, we analyzed cell cultures in the absence of H_2O_2. This revealed that the lack of 6S RNA has little effect on the *E. coli* proteome in the exponential growth phase (Figure S16a), in line with low 6S RNA expression levels at this growth stage (Figure S6). We identified only two proteins in exponential phase: acid phosphatase AphA (dephosphorylates organic phosphate monoesters) and biofilm-promoting protein Flu (antigen 43) whose expression levels were significantly higher in the *ΔssrS* strain, indicating their expression is normally inhibited by 6S RNA in the wild type. In stationary phase, beyond AphA and Flu, also DapA (4-hydroxy-tetrahydrodipicolinate synthase), Mdh (malate dehydrogenase), DppA (dipeptide-binding protein, ABC transporter), Crr (component of sugar phosphotransferase system) and several other proteins were found to be upregulated in the Δ*ssrS* strain (Table S6). We also identified three proteins with higher levels in the wild type: glutamate decarboxylases GadA and GadB and dehydratase FbaA (synthesis of fatty acids).

We observed more differences when comparing WT and *ΔssrS* strains grown in the presence of 5 mM H_2O_2. As expected, this was most evident in the exponential phase (Figure S16b, Table S7), while fewer proteins were affected in stationary phase (Table S8). Notably, in exponential phase at 5 mM H_2O_2 (Table S7), there appeared to be more proteins with higher expression levels in the WT strain (reduced levels in the Δ*ssrS* strain), suggesting indirect 6S RNA-mediated activation through interdigitation with other regulatory pathways (such as utilization of alternative sigma factors by RNAP). This effect vanished in the stationary phase where the *E. coli* proteome seems to be more static regardless of the presence of 6S RNA. All proteins affected by 6S RNA and identified by MALDI-TOF spectroscopy in the exponential phase of *E. coli* growth in the presence of 5 mM H_2O_2 are listed in Tables S7 and S8. Among them we detected two candidates involved in the oxidative stress response: alkyl hydroperoxide reductase AhpC and thiol peroxidase Tpx, which we also identified in our qRT-PCR screen (Figure 6b of the main text). In line with previous findings, expression levels of these two proteins were lower in the Δ*ssrS* strain than in the wild type. Their downregulation may well contribute to the reduced viability of the *ΔssrS* strain under oxidative stress. Other identified proteins participate in different cellular processes, which in general illustrates the function of 6S RNA as a global non-specific regulator of transcription that affects the expression levels of a large set of genes, directly and indirectly.

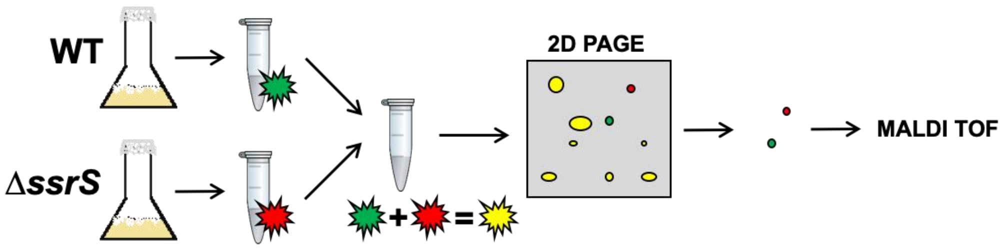

Figure S15. Schematic representation of the comparative proteome analysis workflow. Protein extracts labeled by Cy2 (green, WT) and Cy5 (red, Δ*ssrS*) were mixed in equimolar ratio. As a result, yellow signals corresponded to proteins whose expression was not affected by the lack of 6S RNA. Red or green signals indicate proteins that are activated or inhibited in the Δ*ssrS* strain in comparison to the wild type.

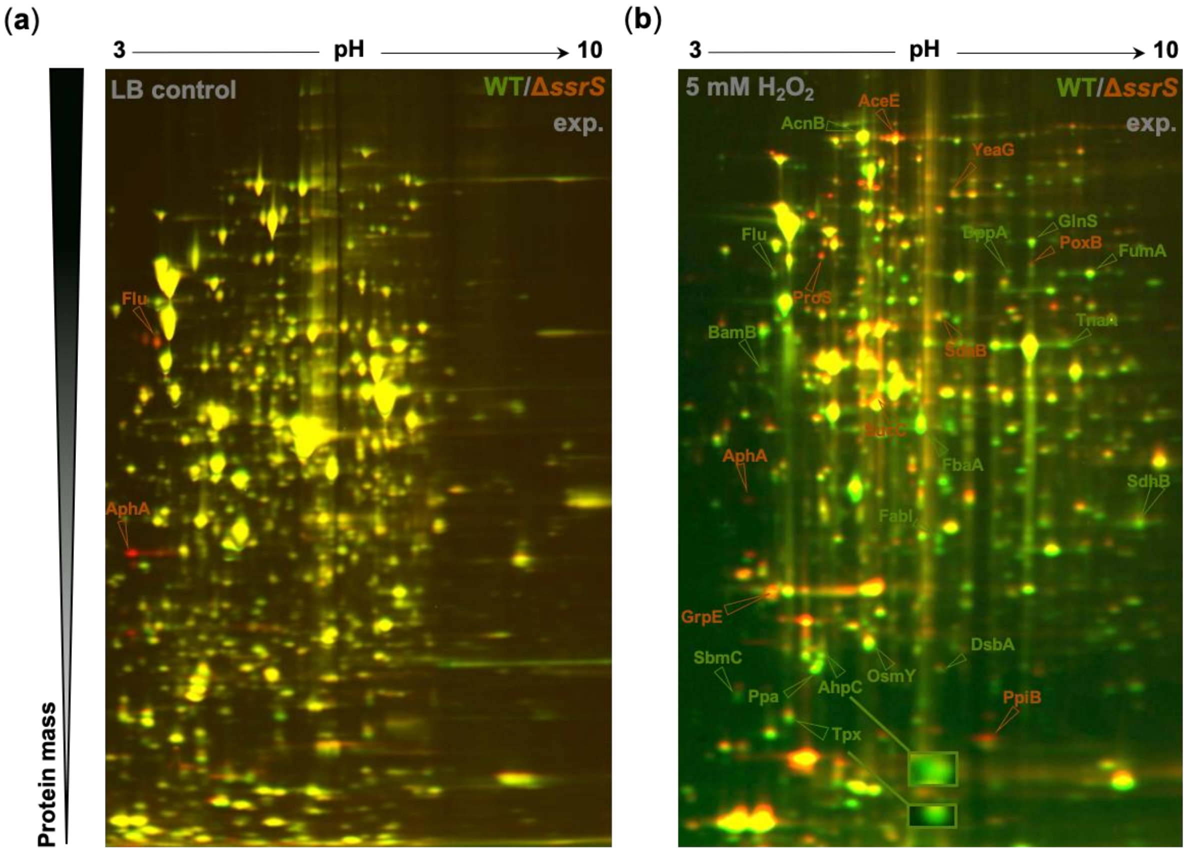

Figure S16. Representative two-dimensional differential gel electrophoresis (2D-DIGE) images of protein extracts from *E. coli* MG1655 WT and the Δ*ssrS* derivative strain; protein extracts were derived from exponential phase cells (OD_{600} = 1) grown under (**a**) standard conditions (LB control without addition of H_2O_2) or (**b**) in the presence of 5 mM H_2O_2. The fluorescence emission images are superimposed in pseudocolors: green for Cy2 (WT) and red for Cy5 (Δ*ssrS*). The horizontal axis depicts a non-linear pH gradient from approx. 3 to 10. Proteins that were identified by MALDI-TOF spectrometry to have altered levels in the Δ*ssrS* strain are indicated.

Table S6. Proteins differentially expressed in *E. coli* MG1655 WT and Δ*ssrS* bacteria in stationary phase under standard conditions of cultivation, identified by MALDI-TOF spectrometry.

Gene	Protein name	Function
	**Elevated in Δ*ssrS* strain**	
aphA	class B acid phosphatase	dephosphorylation of organic phosphate monoesters
flu	antigen 43	surface protein, autotransporter, promotes biofilm formation
dapA	4-hydroxy-tetrahydrodipicolinate synthase	biosynthesis of aminoacids
mdh	malate dehydrogenase	oxydoreductase involved in tricarboxylic acid cycle
dppA	dipeptide-binding protein	part of ABC transporter DppABCDF
crr	PTS system glucose-specific EIIA component	phosphorylation of incoming sugars (including glucose) and translocation across the cell membrane
osmY	osmotically-inducible protein	lipoprotein
hisJ	histidine-binding periplasmic protein	transport of nitrogen compounds
fumA	fumarate hydratase class I	synthesis of metabolites (fumarate and S-malate)
atpA	ATP synthase subunit alpha	ATP synthesis and translocation
lpdA	dihydrolipoyl dehydrogenase	cell redox homeostasis
	**Reduced in Δ*ssrS* strain**	
gadA	glutamate decarboxylase	glutamate metabolism
gadB	glutamate decarboxylase	glutamate metabolism
fabA	3-hydroxydecanoyl-[acyl-carrier-protein] dehydratase	synthesis of fatty acids

Table S7. Proteins differentially expressed in *E. coli* MG1655 WT and Δ*ssrS* bacteria grown to exponential phase in the presence of 5 mM H_2O_2, identified by MALDI-TOF spectrometry.

Gene	Protein name	Function
	**Elevated in Δ*ssrS* strain**	
aceE	pyruvate dehydrogenase E1 component	conversion of pyruvate to acetyl-CoA and CO_2
yeaG	protein kinase	kinase activity, cellular response to nitrogen starvation
poxB	pyruvate dehydrogenase	membrane enzyme, oxidative decarboxylation of pyruvate to acetate and CO_2
proS	proline-tRNA ligase	prolyl-tRNA aminoacylation, attachment of proline to tRNA(Pro)
sdaB	L-serine dehydratase II	conversion of L-serine into pyruvate and ammonia
sucC	succinyl-CoA synthetase subunit β	synthesis of succinyl-CoA
aphA	class B acid phosphatase	dephosphorylation of organic phosphate monoesters
ppiB	peptidyl-prolyl cis-trans isomerase B	cis-trans isomerization of proline imidic peptide bonds in oligopeptides
grpE	nucleotide exchange factor GrpE	release of ADP from DnaK, ATP turnover, hyperosmotic and heat shock response
	**Reduced in Δ*ssrS* strain**	
flu	antigen 43	surface autotransporter, biofilm formation
dppA	dipeptide-binding protein	component of ABC transporter DppABCDF
glnS	glutamine-tRNA ligase	covalently links L-glutamine to its specific tRNA molecule
fumA	fumarate hydratase class I	synthesis of fumarate and S-malate
bamB	outer membrane protein assembly factor	assembly and insertion of beta-barrel proteins into the outer membrane
tnaA	tryptophanase	cleavage of L-tryptophan to indole, pyruvate and NH_4^+
fabI	enoyl-[acyl-carrier-protein] reductase	fatty acid biosynthesis
fbaA	fructose-bisphosphate aldolase class 2	glycolysis, gluconeogenesis and the reverse reactions
sdhB	succinate dehydrogenase subunit	conversion of fumarate and succinate; response to stress-induced mutagenesis
osmY	osmotically-inducible protein	lipoprotein
ppa	inorganic pyrophosphatase	hydrolysis of inorganic pyrophosphate (PPi)
dsbA	thiol:disulfide oxidoreductase	disulfide bond formation in some periplasmic proteins such as PhoA or OmpA
sbmC	DNA gyrase inhibitor	protection of DNA gyrase, response to DNA damage
acnB	aconitate hydratase B	catabolism of short chain fatty acids, nutritional stress response
ahpC	alkyl hydroperoxide reductase C	reduction of H_2O_2 and organic hydroperoxides, oxidative stress response
tpx	thiol peroxidase	reduction of H_2O_2 and organic hydroperoxides, oxidative stress response

* proteins involved in response to oxidative conditions

Table S8. Proteins differentially expressed in *E. coli* MG1655 WT and Δ*ssrS* bacteria grown to stationary phase in the presence of 5 mM H_2O_2, identified by MALDI-TOF spectrometry.

Gene	Protein name	Function
	**Elevated in Δ*ssrS* strain**	
flu	antigen 43	surface autotransporter, promotes biofilm formation
aceE	pyruvate dehydrogenase E1 component	conversion of pyruvate to acetyl-CoA and CO_2
yeaG	protein kinase	kinase activity, cellular response to nitrogen starvation
poxB	pyruvate dehydrogenase	peripheral cell membrane enzyme, catalyzes the oxidative decarboxylation of pyruvate to form acetate and CO_2
glyA	serine hydroxymethyltransferase	converts serine to glycine
mglB	D-galactose-binding periplasmic protein	active transport of galactose and glucose
tcyJ	L-cystine-binding protein TcyJ	component of a cysteine ABC transport system
fabI	enoyl-[acyl-carrier-protein] reductase	fatty acid biosynthesis
adk	adenylate kinase	reversible transfer of the terminal phosphate group between ATP and AMP
wrbA	NAD(P)H dehydrogenase (quinone)	reduces quinones to the hydroquinone state
	**Reduced in Δ*ssrS* strain**	
sbmC	DNA gyrase inhibitor	protection of DNA gyrase, response to DNA damage

References

1. Gößringer, M.; Lechner, M.; Brillante, N.; Weber, C.; Rossmanith, W.; Hartmann, R.K. *Nucleic Acids Res.* **2017**, *45*, 7441-7454. doi: 10.1093/nar/gkx405.
2. Burenina, O.Y.; Elkina, D.A.; Migur, A.Y.; Oretskaya, T.S.; Evguenieva-Hackenberg, E.; Hartmann, R.K.; Kubareva, E.A. Similarities and differences between 6S RNAs from *Bradyrhizobium japonicum* and *Sinorhizobium meliloti*. *J Microbiol*. **2020**, *58*, 945-956. doi: 10.1007/s12275-020-0283-1.
3. Damm, K.; Bach, S.; Müller K.M.; Klug, G.; Burenina, O.Y.; Kubareva, E.A.; Grünweller, A.; Hartmann, R.K.; Impact of RNA isolation protocols on RNA detection by Northern blotting. *Methods Mol Biol*. **2015**, *1296*, 29-38. doi: 10.1007/978-1-4939-2547-6_4.
4. Trotochaud, A.E.; Wassarman, K.M. 6S RNA regulation of *pspF* transcription leads to altered cell survival at high pH, *J. Bacteriol.,* **2006**, *188*, 39363943. doi: 10.1128/JB.00079-06.
